# From Au_11_ to Au_13_: Tailored Synthesis of Superatomic Di-NHC/PPh_3_-Stabilized Molecular Gold Nanoclusters

**DOI:** 10.1021/acs.inorgchem.2c03331

**Published:** 2023-01-13

**Authors:** Matteo Bevilacqua, Marco Roverso, Sara Bogialli, Claudia Graiff, Andrea Biffis

**Affiliations:** † Dipartimento di Scienze Chimiche, Università degli Studi di Padova, Via F. Marzolo 1, 35131Padova, Italy; ‡ Consorzio per le Reattività Chimiche e la Catalisi (CIRCC), c/o Dipartimento di Scienze Chimiche, Università degli Studi di Padova, Via F. Marzolo 1, 35131Padova, Italy; § Dipartimento di Scienze Chimiche, della Vita e della Sostenibilità Ambientale, Università degli Studi di Parma, Parco Area delle Scienze 17/A, 43124Parma, Italy

## Abstract

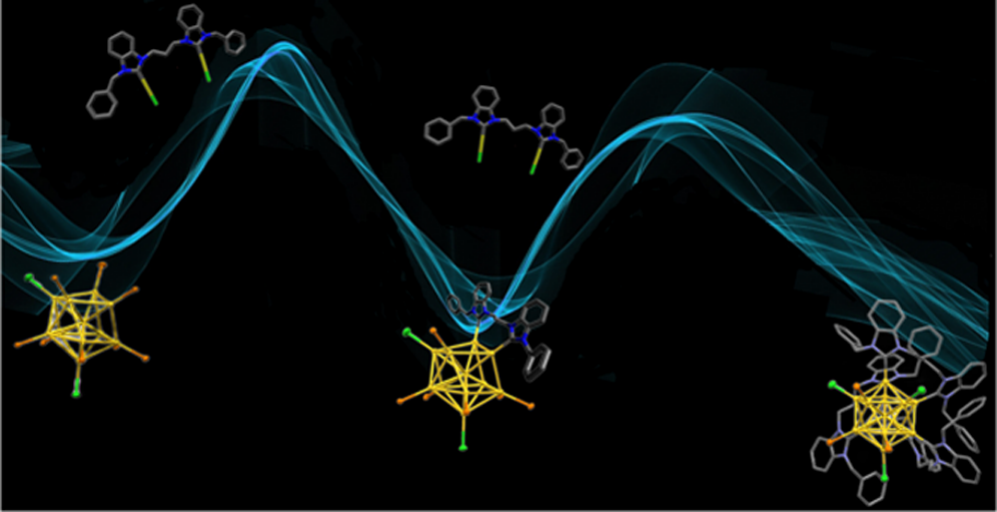

Herein, we report a new method to synthesize molecular gold nanoclusters (AuNCs) stabilized by phosphine (PR_3_) and di-N-heterocyclic carbene (di-NHC) ligands. The interaction of di-NHC gold(I) complexes, with the general formula [(di-NHC)Au_2_Cl_2_] with well-known [Au_11_(PPh_3_)_8_Cl_2_]Cl clusters provides three new classes of AuNCs through a controllable reaction sequence. The synthesis involves an initial ligand metathesis reaction to produce [Au_11_(di-NHC)(PPh_3_)_6_Cl_2_]^+^ (type **1** clusters), followed by a thermally induced rearrangement/metal complex addition with the formation of Au_13_ clusters [Au_13_(di-NHC)_2_(PPh_3_)_4_Cl_4_]^+^ (type **2** clusters). Finally, an additional metathesis process yields [Au_13_(di-NHC)_3_(PPh_3_)_3_Cl_3_]^2+^ (type **3** clusters). The electronic and steric properties of the employed di-NHC ligand affect the product distribution, leading to the isolation and full characterization of different clusters as the main product. A type **3** cluster has been also structurally characterized and was preliminarily found to be strongly emissive in solution.

## Abstract 2

Reaction of digold(I) complexes with N-heterocyclic carbenes and preformed homoleptic phosphine-stabilized molecular gold clusters allows the controllable production of clusters with tailored nuclearity (Au_11_ or Au_13_) and ligand shell composition (di-NHC-to-phosphine ratio), through a reaction sequence involving ligand metathesis and complex addition steps.

## Introduction

Molecular gold nanoclusters (AuNCs) have recently attracted great interest for their structure, consisting of a well-defined metal core stabilized by organic ligands or polynuclear metal complexes, and for their consequent molecular properties.^1−3^ Unlike gold nanoparticles, AuNCs are truly molecular species, characterized by peculiar correlations between their structure, the chemical nature of the stabilizing ligands/complexes, and the resulting molecular properties of the cluster.^4,5^ In particular, the possibility to determine the cluster structure, using single-crystal X-ray diffraction, represents a fundamental tool to get insights into the cluster’s molecular behavior and makes it possible to tailor “ad hoc” AuNCs with specific properties, relevant for applications spanning from catalysis to bio-sensing, bio-imaging, and theranostics.^6−8^

Most studied AuNCs are stabilized by thiolate ligands and are characterized by “staples”, i.e., [Au(I)*_n_*(SR)*_m_*] motifs surrounding the cluster core and stabilizing it.^9−12^ Such staples may impart exceptional stability to AuNCs, which have been synthesized and characterized in several forms, ranging from oligonuclear clusters with metalloid properties up to very high molecular mass metallic species involving hundreds of gold atoms.^13,14^ However, the peculiar staples’ structures in thiolated AuNCs present drawbacks: for example, they complicate ligand substitution and interaction with external molecules, which are of fundamental importance for, e.g., catalytic processes.^6^ Furthermore, the electronic properties of the thiolate ligands cannot be very extensively varied, thus hampering the control over AuNC properties, such as the electronic structure of the cluster, its energy levels, and in ultimate analysis its photophysical behavior.^15^ Consequently, alternative ligand systems are currently being investigated to stabilize AuNCs, such as phosphines (PR_3_),^16−25^ alkynyls,^26−32^ and N-heterocyclic carbenes (NHCs).^32−39^ The latter in particular have been recently shown to be very useful for AuNCs stabilization since very strong Au-NHC bonds can be established.^35,40,41^ Nowadays, a great number of NHC ligands are available, featuring steric and electronic properties that can be extensively and independently varied for this purpose.^42−45^

**Scheme 1:**
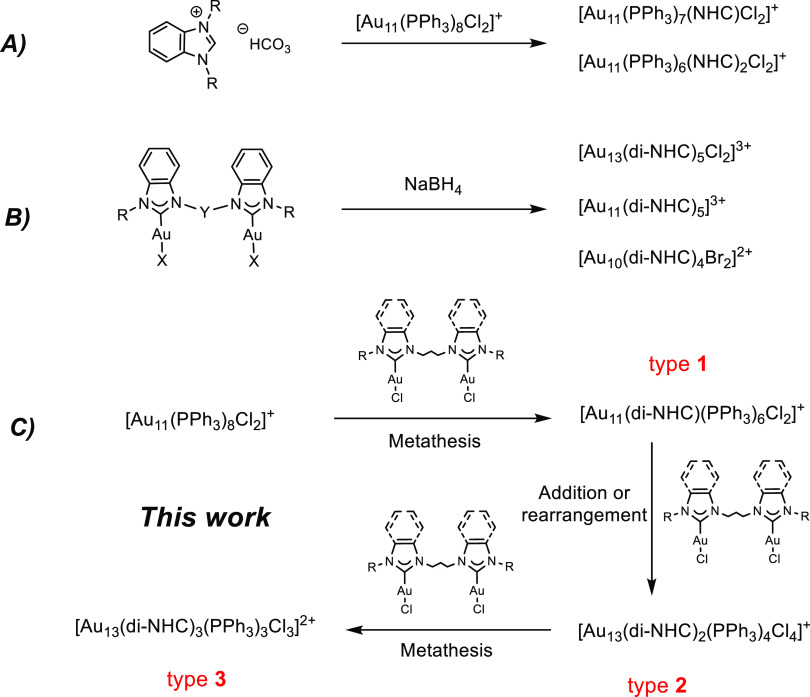
Strategies for the Preparation of NHC-Stabilized AuNCs (A) Substitution reaction with free NHCs formed in situ; (B) direct reduction with NaBH_4_; (C) sequential reaction with Au-NHC complexes (this work).

In 2019, Narouz et al. proved indeed that the insertion by ligand substitution of one NHC ligand on the metalloid cluster [Au_11_(PPh_3_)_8_Cl_2_]Cl (Scheme 1A) improves the cluster stability toward thermal decomposition.^33^ After this work, other AuNCs stabilized only by NHCs or di-NHCs have been synthesized using instead a direct reduction approach (Scheme 1B), in which mono- and di-NHC gold(I) complexes are treated with NaBH_4_ to form Au_11_, Au_13_, Au_25_ and, more recently, Au_10_ clusters.^34−39^ AuNCs with Au cores exhibiting particularly stable electronic configurations (“superatoms”)^46^ are generally produced with the direct reduction approach when NHCs are used as ligands. Among these species, the [Au_13_(NHC)_9_X_3_]^2+^ or [Au_13_(di-NHC)_5_X_2_]^3+^ (X: Br or Cl) icosahedral clusters represent the most common NHC-stabilized AuNCs.^35,36,38,39^ These structures feature peculiar stability imparted by the Au_13_ superatomic core, as well as by the chelating properties of di-NHC ligands.

Our group has a longstanding experience in the chemistry of dinuclear gold(I) complexes with di-NHC ligands,^47−51^ and we envisaged the possibility of exploiting them in an alternative way as reagents for the preparation of novel NHC-stabilized AuNCs (Scheme 1C). Our synthetic approach involves the use of these complexes, which are more stable and easily manipulated than free di-NHCs, for the chemical modification of preformed AuNCs. The use of preformed gold complexes potentially enables two possible reaction pathways with the cluster, namely, ligand metathesis or complex addition reactions. The latter process, formally a “cluster to cluster” synthesis, has been already reported by Mingos et al. in 1996 with PR_3_-protected AuNCs:^52^ they obtained a homoleptic [Au_13_(PR_3_)_8_Cl_4_]^+^ cluster from [Au_11_(PR_3_)_10_]^3+^, using Cl-Au-PR_3_ as a reagent to provide gold atoms and PR_3_ ligands; the reverse process, i.e., gold etching from Au_13_ to Au_11_ cluster using excess phosphine ligands, was also demonstrated.^53^ More recently, it has been proven that reaction of homoleptic thiolate-stabilized AuNCs with gold(I) thiolate precursors bearing the same ligand can result in a “*pseudo*-antigalvanic” reaction of the precursor with the formation of novel homoleptic AuNCs possibly exhibiting a higher or lower number of kernel gold atoms.^54,55^ We now demonstrate that our approach enables the preparation of a library of heteroleptic Au_11_ and Au_13_ clusters protected both by PPh_3_ and di-NHCs, starting from [Au_11_(PPh_3_)_8_Cl_2_]Cl as reagent cluster and reacting it with the digold(I) complexes of general formula [(di-NHC)Au_2_Cl_2_]. Upon changing the stereoelectronic properties of the di-NHC ligand and the reaction conditions, it is possible to control the reaction sequence, which may involve (Scheme 1C) a first metathesis step with formation of [Au_11_(di-NHC)(PPh_3_)_6_Cl_2_]^+^ (type **1** clusters), followed by a thermally induced rearrangement/metal complex addition to get [Au_13_(di-NHC)_2_(PPh_3_)_4_Cl_4_]^+^ (type **2** clusters) and by another metathesis step to obtain [Au_13_(di-NHC)_3_(PPh_3_)_3_Cl_3_]^2+^ (type **3** clusters).

## Experimental Section

All analyses and operations were performed under ambient conditions unless otherwise specified. Benzimidazole, 1-benzyl imidazole, 1,3-dibromopropane, 1,3-dichloropropane, isopropyl bromide, benzyl chloride, potassium carbonate, lithium bromide, lithium chloride, (chloro)dimethylsulfide gold(I), tetraethylammonium chloride monohydrate, and ammonium hexafluorophosphate were purchased from Sigma-Aldrich and used as received. [Au_11_(PPh_3_)_8_Cl_2_]Cl,^33^ 1-benzyl benzimidazole,^56^ 1-isopropyl benzimidazole,^57^ 1,3-di(1*H*-imidazol-yl)propane,^58^ and 1,3-di(1-*H*-benzimidazol-yl)propane^59^ were synthesized according to literature procedures. Tetrahydrofuran (THF), acetonitrile (CH_3_CN), dichloromethane (DCM), dichloroethane (DCE), diethyl ether (Et_2_O), methanol (MeOH), n-hexane, and deuterated solvents were purchased from Sigma-Aldrich. Unless otherwise noted, all solvents were dry and of high purity grade and were used as received.

NMR spectra were recorded on a Bruker Avance 300 MHz (300.1 MHz for ^1^H and 121.5 MHz for ^31^P); chemical shifts (δ) are reported in parts per million (ppm) relative to the residual solvent signals. The multiplicities are reported as follows: singlet (s), doublet (d), triplet (t), quartet (q), quintet (qu), septuplet (st), multiplet (m). The coupling constants (J) are reported in hertz.

The molecular weight and elemental composition of synthesized compounds were analyzed by high-resolution mass spectrometry (HRMS) in flow injection mode (FIA—flow injection analysis). Ten microliters of each sample were injected into the FIA-HRMS system equipped with Agilent 1260 Infinity II LC System coupled to an Agilent 6545 LC/Q-TOF mass analyzer (Agilent Technologies, Palo Alto, CA). The eluent was methanol at 0.5 mL/min flow rate. The MS conditions were: electrospray ionization (ESI) in positive mode, gas temperature 325 °C, drying gas 5 L/min, nebulizer 20 psi, sheath gas temperature 275 °C, sheath gas flow 12 L/min, VCap 4000 V, nozzle voltage 2000 V, fragmentor 180 V. Centroid and profile full scan mass spectra were recorded in the range of 100–10 000 *m*/*z* with a scan rate of 1 spectrum/s. Tandem mass spectrometry (MS/MS) data were acquired in targeted mode with a scan rate of 1 spectrum/s, collision energy of 20 eV, and isolation width of 4 Da. The quadrupole time-of-flight (Q-TOF) calibration was daily performed with the manufacturer’s solution in this mass range. The MS and MS/MS data were analyzed by the Mass Hunter Qualitative Analysis software (Agilent Technologies, Palo Alto, CA).

Absorption spectra were recorded with a PerkinElmer λ950 or λ650 spectrophotometer. All spectra were recorded at room temperature (RT) on 2 × 10^–4^ M cluster solutions in DCM.

Corrected emission spectra were recorded with an Edinburgh photoluminescence spectrometer model FLS1000, with double monochromators, equipped with a 450 W Xe arc lamp as the excitation source. A photomultiplier tube R13456 with a spectral response from 185 to 980 nm was used as a detector. For the emission spectra, the step and dwell time were set at 1 nm and 0.1 s, respectively, and the slit was kept at 0.1 and 3 nm for excitation and emission monochromators, respectively. Fluorescence quantum yields (FQY) were calculated by measuring the emission spectrum of samples dissolved in DCM in a BaSO_4_-coated integration sphere, mounted in the FLS1000 instrument. A cuvette containing DCM solvent was used for the measurement of the reference spectrum. The excitation wavelength was fixed at 350 nm. The emission spectra were recorded in the range of 330–980 nm. The formula used for the calculation of FQY is
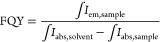
where ∫*I*_em,sample_ is the integrated emission intensity of the sample, and ∫*I*_abs,sample_ and ∫*I*_abs,solvent_ are the integrated absorption of the sample and DCM, respectively.

### Synthesis of Di-Imidazolium Salts

#### Synthesis of 1,1′-Dibenzyl-3,3′-propylene-di-imidazolium Dichloride, [aH_2_]Cl_2_

[**a**H_2_]Cl_2_ salt was synthesized according to a literature procedure.^60^ A two-neck 50 mL balloon was capped with a bubble refrigerator connected with a vacuum line, filled with 1-benzyl imidazole (1.00 g, 6.32 mmol), and sealed with a silicone cap. The apparatus was deareated with three argon–vacuum cycles. 1,3-Dichloropropane (300 μL, 3.15 mmol) and THF (13 mL) were added. The mixture was heated under reflux in an oil bath for 72 h. After this time, the solvent was evaporated under reduced pressure and 10 mL of DCM was added. The resulting white precipitate was filtered and dried under vacuum to afford the product as a white powder. The ^1^H NMR spectra related to the product match with spectra reported in the literature. The solid was used without further purification. Yield 405 mg (15%). ^1^H NMR (400 MHz, CDCl_3_): δ 10.49 (s, 2H), 8.20 (s, 2H), 7.40 (s, 10H), 6.99 (s, 2H), 5.41 (s, 4H), 4.71 (t, *J* = 6.09 Hz, 4H), 2.88 (qu, *J* = 7.22 Hz, 2H).

#### Synthesis of 1,1′-Dibenzyl-3,3′-propylene-dibenzimidazolium Dichloride, [bH_2_]Cl_2_

The following procedure for the preparation of [**b**H_2_]Cl_2_ salt was adapted from the literature.^61^ A two-neck 20 mL balloon was filled with 1,3-di(1-*H*-benzimidazol-yl)propane (510 mg, 1.85 mmol) and sealed with a tap and a silicone cap. The apparatus was deareated with three argon–vacuum cycles. Benzyl chloride (450 μL, 3.91 mmol) and CH_3_CN (3 mL) were added. The mixture was heated at 50 °C in an oil bath for 72 h. After 24 h, it was possible to notice the formation of a white precipitate. After 72 h, this precipitate was filtered and washed with 2 × 10 mL Et_2_O. The solid was dried under vacuum to afford the product as a white powder. The ^1^H NMR spectra related to the product match with the spectra reported in the literature with bromide anions. The solid was used without further purification. Yield 636 mg (65%). ^1^H NMR (400 MHz, dimethyl sulfoxide (DMSO)-*d*_6_): δ 10.50 (s, 2H), 8.22 (d, *J* = 7.44 Hz, 2H), 7.97 (d, *J* = 8.10 Hz, 2H), 7.78–7.573 (m, 8H), 7.47–7.32 (m, 6H), 5.84 (s, 4H), 4.79 (t, *J* = 7.59 Hz, 4H), 2.70 (qu, *J* = 7.61 Hz, 2H).

#### Synthesis of 1,1′-Diisopropyl-3,3′-propylene-dibenzimidazolium Dibromide, [cH_2_]Br_2_

The following procedure for the preparation of [**c**H_2_]Br_2_ salt was adapted from the literature.^57^ A two-neck 20 mL balloon capped with a bubble refrigerator was filled with 1-isopropyl benzimidazole (243 mg, 1.52 mmol). The apparatus was deareated with three argon–vacuum cycles. 1,3-Dibromopropane (70 μL, 0.67 mmol) and CH_3_CN (3 mL) were added. The mixture was heated at 90 °C in an oil bath for 72 h. After 24 h, it was possible to notice the presence of a white precipitate. After 72 h, the white precipitate was filtered and washed with 3 × 10 mL toluene and 2 × 10 mL Et_2_O. The solid was dried under vacuum to afford the product as a white powder. The ^1^H NMR spectra related to the product match with the spectra reported in the literature. The solid was used without further purification. Yield 201 mg (83%). ^1^H NMR (400 MHz, CDCl_3_): δ 11.06 (s, 2H), 8.78 (d, *J* = 8.13 Hz, 2H), 7.83–7.53 (m, 6H), 5.20 (t, *J* = 7.75 Hz, 4H), 4.89 (qu, *J* = 6.39 Hz, 2H), 3.08 (st, *J* = 7.70 Hz, 2H), 1.82 (d, *J* = 6.98 Hz, 12H).

#### Synthesis of [cH_2_](PF_6_)_2_

A one-neck 10 mL balloon was filled with [**c**H_2_]Br_2_ (400 mg, 0.77 mmol) and 1.5 mL of MeOH and was stoppered with a silicone cap. An aqueous solution of NH_4_PF_6_ (428 mg, 2.32 mmol, in 1.5 mL of H_2_O) was prepared and added dropwise on the solution containing the imidazolium salt. The mixture was left under stirring for 2 h. The resulting white precipitate was filtered and washed with 2 × 5 mL 1:1 MeOH/H_2_O solution. The solid was dried under vacuum to afford the product as a white powder. Yield 441 mg (88%). ^1^H NMR (400 MHz, CD_3_CN): δ 9.19 (s, 2H), 7.98–7.91 (m, 4H), 7.76–7.73 (m, 4H), 5.00 (qu, *J* = 7.29 Hz, 2H), 4.61 (t, *J* = 67.49 Hz, 4H), 2.71 (st, *J* = 7.50 Hz, 2H), 1.71 (d, *J* = 6.66 Hz, 12H).

### Synthesis of Di-NHC Gold(I) Complexes

#### Synthesis of **c-Br**

A two-neck 25 mL balloon was filled with (chloro)dimethylsulfide gold(I) (41 mg, 0.14 mmol), [**c**H_2_]Br_2_ (36 mg, 0.070 mmol), K_2_CO_3_ (261 mg, 1.89 mmol), and LiBr (42 mg, 0.48 mmol). The balloon was stoppered with a tap and a silicone cap. The apparatus was deareated with three argon–vacuum cycles and CH_3_CN (5 mL) was added. The mixture was heated at 60 °C in an oil bath for 16 h. The mixture was filtered and washed with 2 × 10 mL CH_3_CN. The filtrate and washing solutions were combined, and the solvent was evaporated under vacuum to obtain a green solid residue. The residue was dispersed in 5 mL of DCM, and the obtained solution was filtered through a poly(tetrafluoroethylene) (PTFE) filter. The solvent was evaporated under reduced pressure, and the solid residue was taken up in 1 mL of DCM. The DCM solution was added dropwise to 25 mL of *n*-hexane to produce a white precipitate. The solid was filtered and dried under vacuum to afford the product as a white powder. Yield 48.4 mg (78%). ^1^H NMR (400 MHz, CDCl_3_): δ 7.75–7.60 (m, 4H), 7.57–7.40 (m, 4H), 5.42 (qu, *J* = 6.50 Hz, 2H), 4.68 (t, *J* = 6.96 Hz, 4H), 2.73 (st, *J* = 8.05 Hz, 2H), 1.74 (d, *J* = 6.72 Hz, 12H). Elemental analysis calcd (%) for C_23_H_28_N_4_Br_2_Au_2_: C 30.22, H 3.09, N 6.13; found: C 30.09, H 3.21, N 5.95.

#### Synthesis of **a**

A two-neck 25 mL balloon was filled with (chloro)dimethylsulfide gold(I) (69 mg, 0.24 mmol), [**a**H_2_]Cl_2_ (50 mg, 0.12 mmol), K_2_CO_3_ (320 mg, 2.31 mmol), LiCl (50 mg, 1.2 mmol), and stoppered with a tap and a silicon cap. The apparatus was deareated with three argon–vacuum cycles and CH_3_CN (3 mL) was added. The mixture was heated at 60 °C in an oil bath for 16 h. The mixture was filtered and washed with 2 × 10 mL CH_3_CN. The filtrate and washing solutions were combined and concentrated under vacuum to ca. 1 mL. Et_2_O (25 mL) was then added dropwise to the DCM solution to produce a white precipitate. The solid was filtered and dried under vacuum to afford the product as a white powder. Yield 52.6 mg (44%). ^1^H NMR (400 MHz, CDCl_3_): δ 7.37 (m, 10H), 7.07 (d, *J* = 1.80 Hz, 2H), 6.93 (d, *J* = 1.81 Hz, 2H), 5.44 (s, 4H), 4.28 (t, *J* = 6.50 Hz 4H), 2.51 (qu, *J* = 6.93 Hz, 2H). Elemental analysis calcd (%) for C_23_H_24_N_4_Cl_2_Au_2_: C 33.64, H 2.95, N 6.82; found: C 33.30, H 3.12, N 6.68.

#### Synthesis of **b**

A two-neck 25 mL balloon was filled with (chloro)dimethylsulfide gold(I) (72 mg, 0.24 mmol), [**b**H_2_]Cl_2_ (63 mg, 0.12 mmol), K_2_CO_3_ (320 mg, 2.31 mmol), and LiCl (50 mg, 1.2 mmol) and stoppered with a tap and a silicon cap. The apparatus was deareated with three argon–vacuum cycles and CH_3_CN (3 mL) was added. The mixture was heated in 60 °C in an oil bath for 16 h. The mixture was filtered and washed with 2 × 10 mL CH_3_CN. The complex was extracted from the filtered solid using 10 mL of DCM. The resulting DCM solution was subsequently concentrated to 1 mL under vacuum. Et_2_O (25 mL) was added dropwise to produce a white precipitate. The solid was dried under vacuum to afford the product as a white powder. Yield 73.7 mg (69%). ^1^H NMR (400 MHz, CDCl_3_): δ 7.60–7.24 (m, 18H), 5.78 (s, 4H), 4.71 (t, *J* = 7.66 Hz, 4H), 2.82 (q, *J* = 7.67 Hz, 2H). Elemental analysis calcd (%) for C_31_H_28_N_4_Cl_2_Au_2_: C 40.41, H 3.06, N 6.08; found: C 40.21, H 3.50, N 5.89.

#### Synthesis of **c**

A two-neck 25 mL balloon was filled with (chloro)dimethylsulfide gold(I), (145 mg, 0.49 mmol), [**c**H_2_](PF_6_)_2_ (150 mg, 0.23 mmol), K_2_CO_3_ (696 mg, 5.04 mmol), and NEt_4_Cl·H_2_O (91 mg, 0.50 mmol) and stoppered with a tap and a silicon cap. The apparatus was deareated with three argon–vacuum cycles and CH_3_CN (8 mL) was added. The mixture was heated at 60 °C in an oil bath for 16 h. The mixture was filtered and washed with 2 × 10 mL CH_3_CN. The filtrate and washing solutions were combined, and the solvent was evaporated under vacuum to obtain a green solid residue. MeOH (40 mL) was added, and the resulting mixture was left under stirring for 10 min. The insoluble solid was filtered and dried under reduced pressure to afford the product as a light brown solid. Yield 37.3 mg (20%). ^1^H NMR (400 MHz, CDCl_3_): δ 7.66–7.57 (m, 4H), 7.55–7.37 (m, 4H), 5.44 (q, *J* = 6.57 Hz, 2H), 4.66 (t, *J* = 6.75 Hz, 4H), 2.71 (st, *J* = 7.94 Hz, 2H), 1.75 (d, *J* = 6.76 Hz, 12H). Elemental analysis calcd (%) for C_23_H_28_N_4_Cl_2_Au_2_: C 33.47, H 3.42, N 6.79; found: C 33.68, H 3.71, N 7.01.

### Synthesis of AuNCs

All reactions were performed in air using DCM as solvent. All reactions were monitored using quadrupole time-of-flight high-resolution mass spectrometry (Q-TOF HRMS).

### Experiment with 1 equiv of Gold(I) Complex

[Au_11_(PPh_3_)_8_Cl_2_]Cl (5.8 mg, 1.3 × 10^–3^ mmol) and **c-Br** (1.2 mg; 1.3 × 10^–3^ mmol) were weighed in two different vials. The solids were each dissolved in 0.25 mL of DCM to afford a total volume of solution equal to 0.5 mL. The obtained solutions were mixed and stirred at room temperature for 31 days. After 21 days, Q-TOF HRMS high mass spectrometry highlighted the presence of the **1c** cluster. After 31 days, the mixture was placed in a 40 °C oil bath and left under stirring for 16 h. After overnight warming, Q-TOF HRMS mass spectrometry highlighted the presence of the **2c** cluster. The mixture was further stirred at room temperature for another 15 days, after which it was possible to observe degradation of the solution with the formation of a black precipitate.

### Experiments with 2 equiv of Gold(I) Complex

#### Experiment with Complex **a**: **2a** Cluster Synthesis

[Au_11_(PPh_3_)_8_Cl_2_]Cl (4.0 mg, 0.92 × 10^–3^ mmol) and **a** (1.5 mg, 1.8 × 10^–3^ mmol) were weighed in two different vials. The solids were each dissolved in 0.75 mL of DCM, to afford a total volume of solution equal to 1.50 mL. The obtained solutions were mixed and left under stirring at 40 °C for 16 h. Subsequently, the mixture was stirred at room temperature for 21 days. After this time, Q-TOF HRMS mass spectrometry highlighted the presence of clusters **2a** and **3a**. The solvent was evaporated under reduced pressure and 0.75 mL of DCM was added. The sample underwent crystallization upon condensation of Et_2_O vapor at −4 °C: after one night, a pale orange solution and a deep red solid on the vial bottom were present. The solution was discharged, and the red solid was dispersed in 1.40 mL of DCE. The solution was filtered on a PTFE filter and placed under Et_2_O vapor at room temperature for 1 day, causing again the separation of a deep red solid. The solution was removed, and the red solid residue was dissolved in 0.3 mL of DCM. The solution was loaded on a chromatographic silica column and eluted using DCM/MeOH (9/1) as eluent (Rf_**2a**_: 0.68). Orange fractions were collected, and solvent evaporation afforded cluster **2a** as a red solid. Yield 0.9 mg (22%). Q-TOF HRMS (*m*/*z*): 4463.3348 ([Au_13_(di-NHC^a^)_2_(PPh_3_)_4_Cl_4_]^+^), 2243.1628 *m*/*z* ([Au_13_(di-NHC^a^)_2_(PPh_3_)_4_Cl_4_](Na)^2+^); ^31^P NMR (400 MHz, CD_2_Cl_2_): δ 57.11 (q, *J* = 5.04 Hz, 1P), δ 53.52 (q, *J* = 5.03 Hz, 1P), δ 51.09 (q, *J* = 5.05 Hz, 1P), δ 50.79 (q, *J* = 5.04 Hz, 1P).

#### Experiment with Complex **b**: **3b** Cluster Synthesis

[Au_11_(PPh_3_)_8_Cl_2_]Cl (4.0 mg, 0.92 × 10^–3^ mmol) and **b** (1.7 mg, 1.9 × 10^–3^ mmol) were weighed in two different vials. The solids were each dissolved in 0.75 mL of DCM, to afford a total volume of solution equal to 1.50 mL. The obtained solutions were mixed and left under stirring at 40 °C for 16 h. Subsequently, the mixture was stirred at room temperature for 17 days. After this time, Q-TOF HRMS highlighted the presence of **2b** and **3b** clusters. The solvent was evaporated, and 0.75 mL of DCM was added. The sample underwent crystallization upon condensation of Et_2_O vapor at room temperature. After 1 day, a pale orange solution and deep red solid on the vial bottom were present. The solution was removed with a Pasteur pipette, and the red solid was dried under reduced pressure. The solid was dispersed in 1.00 mL of DCE. The obtained mixture was filtered on a PTFE filter and placed under Et_2_O vapor at room temperature for 1 day, causing again the separation of a deep red solid. The solution was removed, and the red solid was dissolved in 0.3 mL of DCM-*d*_2_. Both Q-TOF HRMS and ^31^P NMR highlighted the presence of clusters **2b** and **3b**. The mixture was loaded on a chromatographic silica column and eluted using DCM/MeOH (9/1) as eluent (Rf_**3b**_: 0.30). Orange fractions were collected, and solvent evaporation afforded cluster **3b** as a red solid. Yield 1.7 mg (40%). Q-TOF HRMS (*m*/*z*): 2411.7340 ([Au_13_(di-NHC^b^)_3_(PPh_3_)_3_Cl_3_]^2+^); ^31^P NMR (400 MHz, CD_2_Cl_2_): δ 60.83 (s, 3P).

#### Second Experiment with Complex **b**: **1b** Cluster Synthesis

[Et(PPh_3_)_8_Cl_2_]Cl (19.6 mg, 4.5 × 10^–3^ mmol) and **b** (9.0 mg, 9.0 × 10^–3^ mmol) were weighed in two different vials. The solids were each dissolved in 4 mL of DCM, to afford a total volume of solution equal to 8 mL. The obtained solutions were mixed and left under stirring at 40 °C for 72 h. The obtained mixture was filtered on a PTFE filter, loaded on a chromatographic silica column and eluted, using DCM/MeOH (9/1) as eluent (Rf_**1b**_: 0.40). Orange fractions were collected, and solvent evaporation afforded cluster **1b** as a red solid. Yield 2.3 mg (12%). Q-TOF HRMS (*m*/*z*): 4267.7004 ([Au_11_(di-NHC^b^)(PPh_3_)_6_Cl_2_]^+^), 2067.6816 ([Au_11_(di-NHC^b^)(PPh_3_)_6_Cl]^2+^); ^31^P NMR (400 MHz, CD_2_Cl_2_): δ 52.31 (s, 6P).

#### Experiment with Complex **c**

[Au_11_(PPh_3_)_8_Cl_2_]Cl (4.0 mg, 0.92 × 10^–3^ mmol) and **c** (1.5 mg; 1.8 × 10^–3^ mmol) were weighed in two different vials. The solids were each dissolved in 0.75 mL of DCM, to afford a total volume of solution equal to 1.50 mL. The obtained solutions were mixed and placed under stirring at 40 °C for 16 h. Subsequently, the mixture was stirred at room temperature for 40 days. After 32 days, Q-TOF HRMS highlighted the presence of cluster **1c**, together with smaller amounts of clusters **2c** and **3c**. After 45 days, it was possible to observe the degradation of the solution with the formation of a black precipitate.

#### Second Experiment with Complex **c**: **1c** Cluster Synthesis

[Au_11_(PPh_3_)_8_Cl_2_]Cl (16.2 mg; 4.05 × 10^–3^ mmol) and **c** (6.1 mg; 7.40 × 10^–3^ mmol) were weighed in two different vials. The solids were each dissolved in 3.25 mL of DCM, to afford a total volume of solution equal to 6.50 mL. The obtained solutions were mixed and placed under stirring at 40 °C for 3 days. The solution was subsequently filtered on a PTFE filter, and the solvent was evaporated at reduced pressure; 1 mL of DCM was then added to the red residue. The sample underwent overnight crystallization upon condensation of Et_2_O vapor at room temperature. The orange solution derived from crystallization was collected, concentrated, and loaded on a chromatographic silica column, using DCM/MeOH (9/1) as eluent. All orange fractions were collected, and solvent evaporation afforded cluster **1c** as a red solid. In this case, a trace of cluster [Au_11_(PPh_3_)_8_Cl_2_]Cl was detected in the final sample; exploiting ^31^P NMR spectrum integrals, it is possible to estimate the purity of cluster **1c** to be around 99%. Yield 3.7 mg (22%). Q-TOF HRMS (*m*/*z*): 4171.3448 ([Au_11_(di-NHC^c^)(PPh_3_)_6_Cl_2_]^+^). ^1^H NMR (400 MHz, CD_2_Cl_2_): δ 7.48–7.29 (m, 57H), 7.03–6.94 (m, 17H), 6.89–6.79 (m, 24H), 6.20 (br, 2H), 4.10 (br, 4H), 3.00 (br, 2H), 0.92 (d, *J* = 6.15 Hz, 12H). ^31^P NMR (400 MHz, CD_2_Cl_2_): δ 52.71 (br, 5P), 51.37 (br, 1P).

### Experiments with 3 equiv of Gold(I) Complex

#### Experiment with Complex **a**

[Au_11_(PPh_3_)_8_Cl_2_]Cl (3.8 mg; 0.87 × 10^–3^ mmol) and **a** (2.1 mg; 2.6 × 10^–3^ mmol) were weighed in two different vials. The solids were each dissolved in 0.75 mL of DCM, to afford a total volume of solution equal to 1.50 mL. The obtained solutions were mixed and placed under stirring at 40 °C for 24 h. After this time a white precipitate was noticed in solution and filtered. Q-TOF HRMS of the orange solution highlighted the presence of clusters **2a** and **3a** in traces only, together with [Au_2_(di-NHC^a^)_2_]^2+^, detected at 553 *m*/*z*.

#### Experiment with Complex **b**: **2b** and **3b** Cluster Synthesis

[Au_11_(PPh_3_)_8_Cl_2_]Cl (20.2 mg; 4.60 × 10^–3^ mmol) and **b** (12.8 mg; 13.9 × 10^–3^ mmol) were weighed in two different vials. The solids were each dissolved in 4.00 mL of DCM, to afford a total volume of solution equal to 8.00 mL. The obtained solutions were mixed and placed under stirring at 40 °C for 3 days. After this time, Q-TOF HRMS highlighted the presence of clusters **2b** and **3b**. The reaction mixture was directly loaded on a chromatographic silica column and eluted using DCM/MeOH (9/1). The first orange fraction (Rf: 0.60) was collected and set aside. The second orange fraction (Rf: 0.30) was collected, and the solvent was evaporated under reduced pressure to afford **3b** as a red solid. Yield 5.0 mg (22%). The first orange fraction was evaporated to dryness to obtain a red solid, which was redissolved in 0.50 mL of DCM. This solution was again loaded on a chromatographic silica column and eluted using the same eluent as before. The first orange fraction (Rf: 0.60) was collected, and solvent evaporation under reduced pressure afforded cluster **2b** as a red solid. Yield 2.3 mg (11%). **2b**: Q-TOF HRMS (*m*/*z*): 4664.3051 ([Au_13_(di-NHC^b^)_2_(PPh_3_)_4_Cl_4_]^+^), 2343.6454 *m*/*z* ([Au_13_(di-NHC^b^)_2_(PPh_3_)_4_Cl_4_](Na)^2+^), ^31^P NMR (400 MHz, CD_2_Cl_2_): δ 27.45 (s, 4P). **3b**: Q-TOF HRMS (*m*/*z*): 2411.7408 ([Au_13_(di-NHC^b^)_3_(PPh_3_)_3_Cl_3_]^2+^) ^31^P NMR (400 MHz. CD_2_Cl_2_): δ 60.83 (s, 3P).

#### Second Experiment with Complex **b**: **3b** Cluster Synthesis

[Au_11_(PPh_3_)_8_Cl_2_]Cl (19.8 mg; 4.51 × 10^–3^ mmol) and **b** (12.6 mg; 13.68 × 10^–3^ mmol) were weighed in two different vials. The solids were each dissolved in 4.00 mL of DCM, to afford a total volume of solution equal to 8.00 mL. The obtained solutions were mixed and placed under stirring at 40 °C for 5 days. After this time, Q-TOF HRMS highlighted the presence of cluster **3b**. The reaction mixture was directly loaded on a chromatographic silica column and eluted using DCM/MeOH (9/1) as eluent. The orange fraction was collected, and the solvent was evaporated under reduced pressure to afford **3b** as a red solid. Yield 7.1 mg (32%). Q-TOF HRMS (*m*/*z*): 2411.7408 ([Au_13_(di-NHC^b^)_3_(PPh_3_)_3_Cl_3_]^2+^). ^31^P NMR (400 MHz; CD_2_Cl_2_): δ 60.83 (s, 3P).

## Results and Discussion

**Figure 1:**
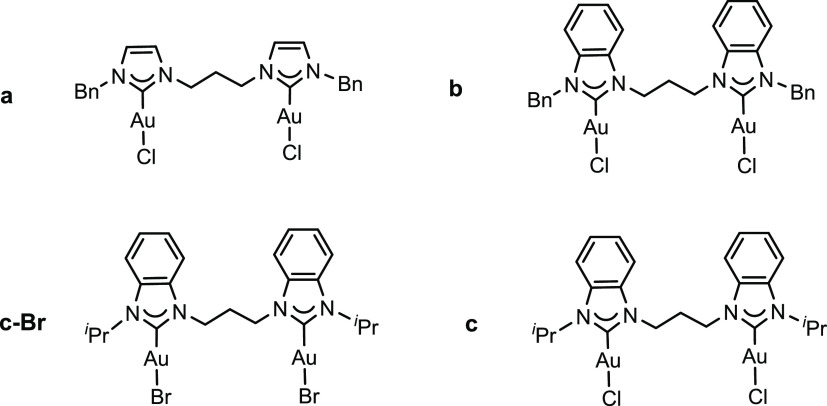
Employed di-NHC digold(I) complexes.

We started by preparing through literature methods or slight variations thereof the neutral complexes **a**, **b**, **c**, and **c-Br** (Figure 1), differing for the nature of the carbene heterocycle, of the wingtip substituents and of the halide ligands. We maintained in all of these complexes a 1,3-propylene bridge between the carbene units, since in our experience, it ideally matches the length and flexibility to bridge two mutually interacting gold centers.^62^ The digold(I) complexes were then all reacted with the well-known phosphine cluster [Au_11_(PPh_3_)_8_Cl_2_]Cl in dry dichloromethane under different sets of reaction conditions. Q-TOF high-resolution mass spectrometry was routinely employed to monitor the reaction progress.

**Figure 2:**
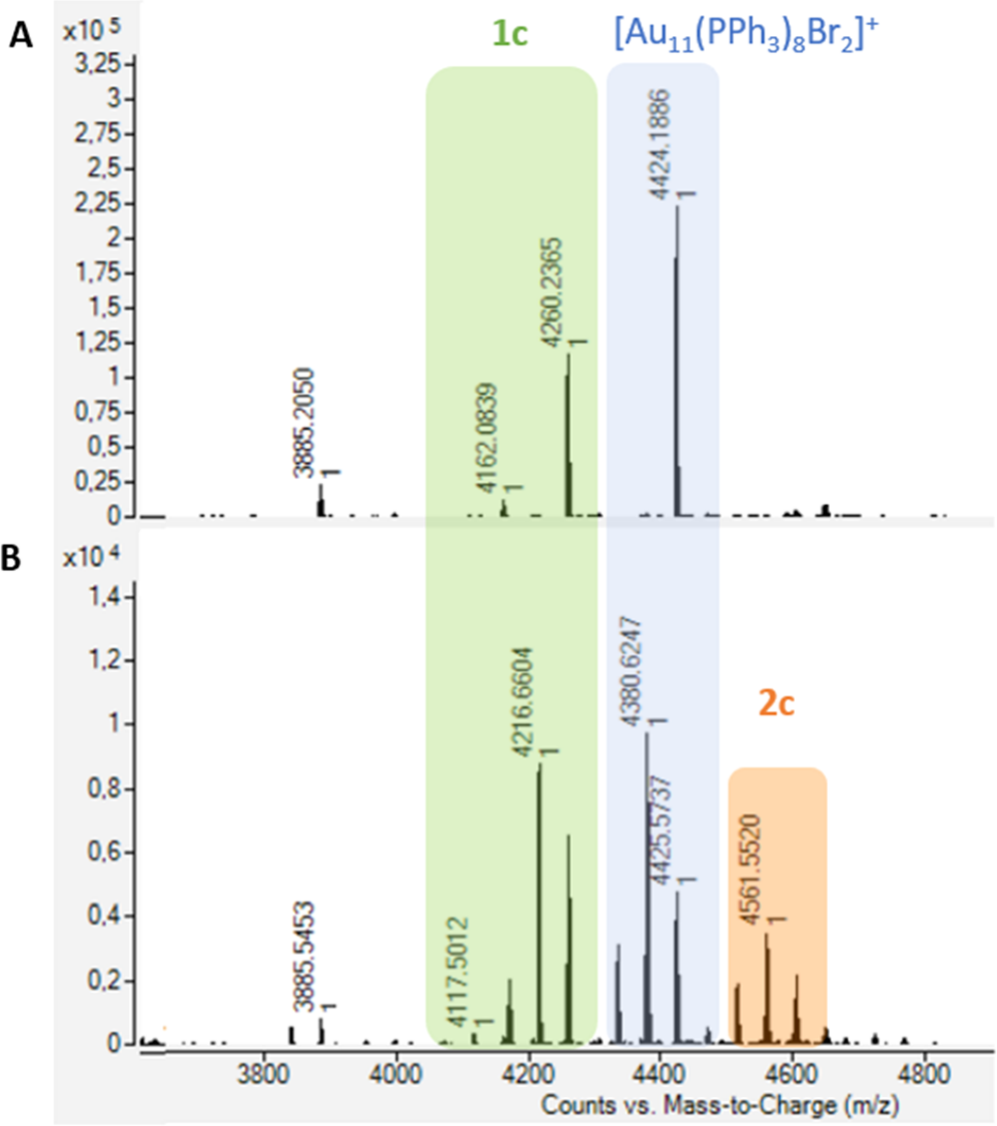
Q-TOF analyses of the reaction of [Au_11_(PPh_3_)_8_Cl_2_]Cl with complex **c-Br** after 21 days at RT (A) and after warming at 40 °C (B). Color code for signal identification: [Au_11_(PPh_3_)_8_Cl_2_]^+^ blue, **1c** green, **2c** orange. Multiple signals in (B) arise from extensive halide scrambling taking place at 40 °C.

The first experiment was performed at room temperature with a 1:1 molar ratio between [Au_11_(PPh_3_)_8_Cl_2_]Cl and the **c-Br** complex. In Figure 2, the corresponding Q-TOF analyses are reported. A very slow metathesis reaction was found to take place: after 21 days the appearance of a signal at 4260 *m*/*z* corresponding to **1c**, with the formula [Au_11_(di-NHC^c^)(PPh_3_)_6_Br_2_]^+^, was recorded, together with the mononuclear complex Ph_3_PAuBr as the expected metathesis coproduct. After additional 10 days, it became clear that the reaction was not proceeding further; consequently, overnight warming was used to speed up the process. After 16 h at 40 °C, conversion of the original cluster to **1c** increased and the formation of a new cluster, named **2c**, was identified at 4500–4600 *m*/*z*, with general stoichiometric formula [Au_13_(di-NHC^c^)_2_(PPh_3_)_4_X_4_]^+^ (X: Cl and/or Br). Apparently, the new Au_13_ cluster derives from a metal complex addition that occurs on **1c**. It must be also remarked that after heating at 40 °C each cluster species gave rise to multiple signals in the Q-TOF mass spectrum due to extensive halide scrambling between chloride and bromide.

The result of this experiment demonstrates for the first time that a slow metathesis reaction between a phosphine-stabilized AuNC and di-NHC digold(I) complex is feasible and leads to the first example of a mixed ligand Au_11_ cluster bearing phosphines and a di-NHC ligand (previously, only mixed ligand clusters with mono-NHCs have been prepared). Remarkably, it also shows for the first time that a slight temperature increase can trigger a further reaction, with the production of a mixed ligand Au_13_ cluster. The experiment also highlighted some drawbacks of the procedure, namely, the very slow rate of the metathesis process at the employed reaction temperature and the extensive scrambling of halide ligands in the clusters, which called for the use of starting materials bearing all the same halide. Consequently, use of complex **c-Br** as a reagent was discontinued in favor of complexes **a**–**c**.

**Figure 3:**
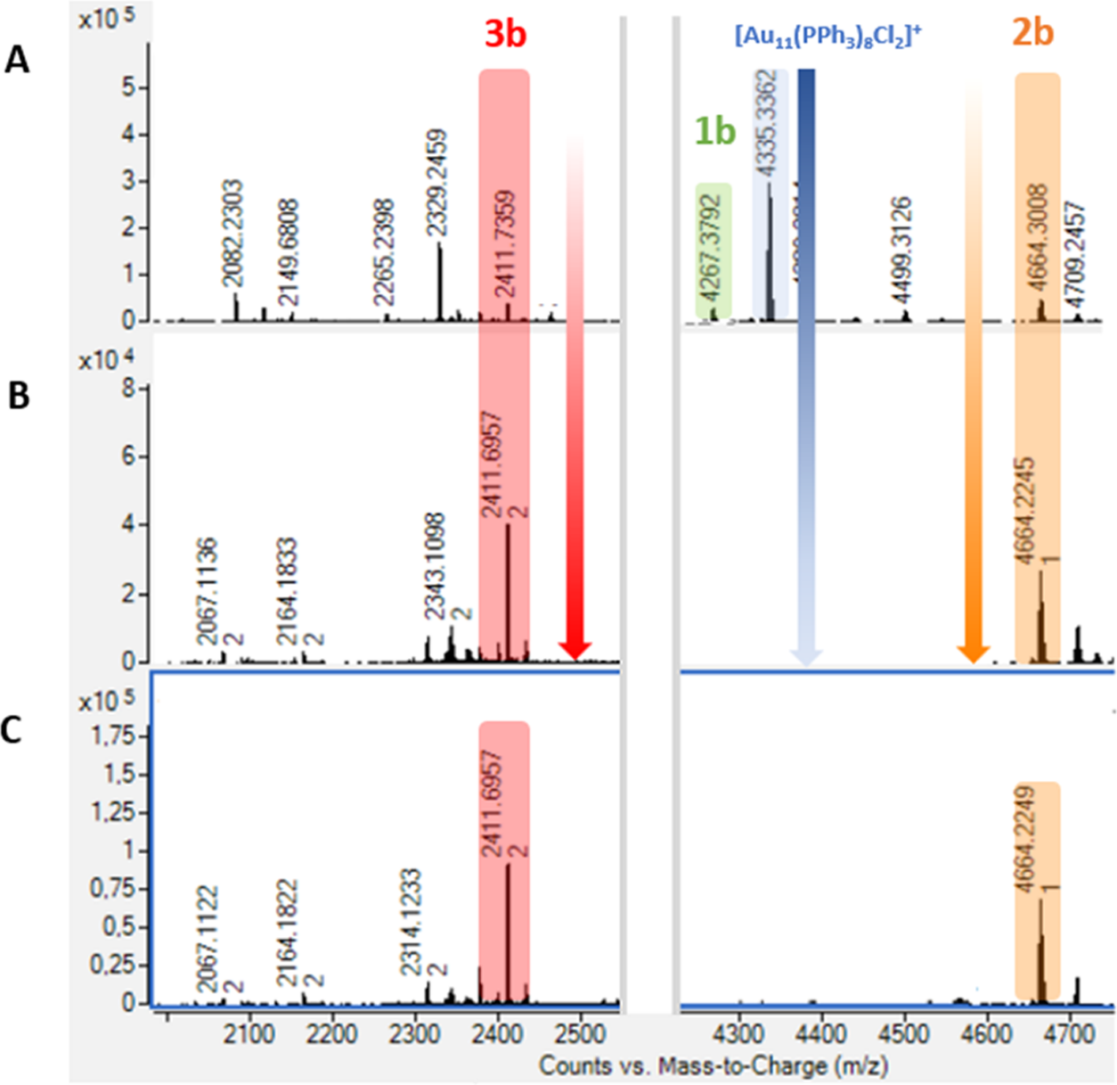
Q-TOF analyses of the reaction of [Au_11_(PPh_3_)_8_Cl_2_]Cl with 2 equiv of complex **b**. From above, after overnight warming (A) and after 17 days at RT (B). In (C), the signals of the produced clusters after purification.

In a second set of experiments, we directly exploited overnight heating of the reaction mixture at 40 °C, to shorten the reaction time. All three complexes **a**, **b**, and **c** were evaluated to explore the generality of the synthetic approach and the effect of the different di-NHC ligands on the resulting AuNCs. A molar ratio [Au_11_]/[(di-NHC)Au_2_Cl_2_] equal to 1:2 was also employed, to promote the formation of Au_13_ clusters. After 16 h heating at 40 °C, the formation of type **1** clusters was detected with all complexes, together with type **2** clusters. Surprisingly, in-depth Q-TOF analysis highlighted, in the case of complexes **a** and **b**, also the presence of a third type of Au_13_ cluster, namely, type **3** clusters **3a** and **3b** (Figure 3). These latter AuNCs are centered, respectively, at 2261 *m*/*z* and 2411 *m*/*z*, corresponding to [Au_13_(di-NHC)_3_(PPh_3_)_3_Cl_3_]^2+^ stoichiometry and therefore featuring a third di-NHC ligand bonded to the metallic core. The formation of such type **3** clusters is likely the result of an additional metathesis that takes place between the complexes and the type **2** clusters. It needs to be remarked that after overnight heating, the conversion of the reagent cluster [Au_11_(PPh_3_)_8_Cl_2_]Cl to mixed ligand clusters is in all cases only partial, indicating that the first metathesis process proceeds at a rate comparable to subsequent reactions, at least for what it concerns reactions with complexes **a** and **b**.

Indeed, in the experiments involving **a** and **b**, the reagent cluster is consumed within further 21 and 17 days at room temperature, respectively; within the same time, clusters **1a** and **1b** disappear completely, yielding in both experiments a mixture of Au_13_ clusters of type **2** and **3**. In the experiment involving **c**, cluster **1c** persists instead for more than 30 days in solution, only very slowly and partially converting to Au_13_ clusters. In Figure 3, the Q-TOF analyses at all stages related to the experiment made with **b** are reported. The Q-TOF analyses related with the same experiment made with **a** and **c** are reported in Figures S26 and S32, Supporting Information, respectively.

**Figure 4:**
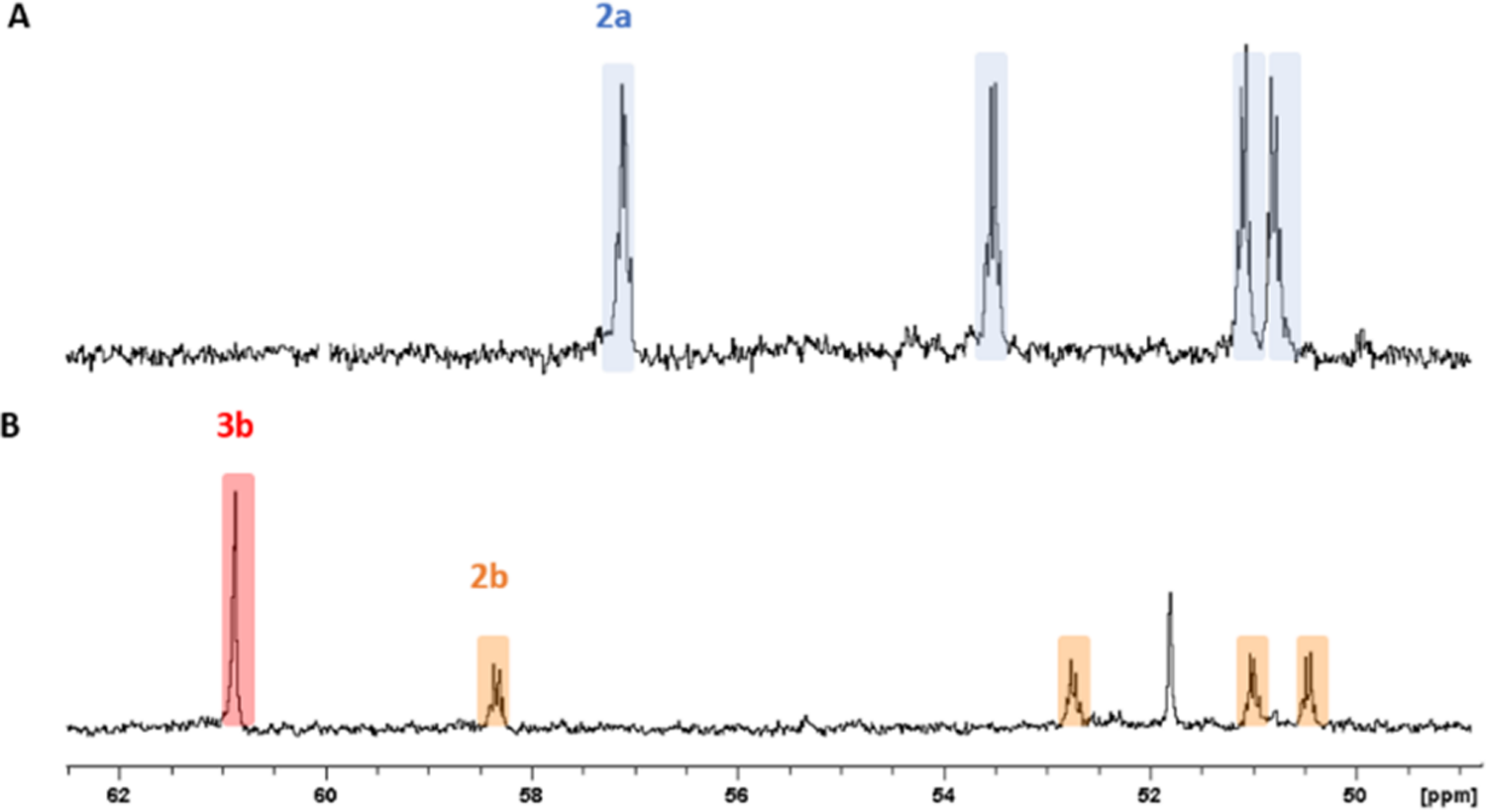
^31^P NMR spectra of cluster **2a** (A) and of the crude mixture of clusters **2b** (orange) and **3b** (red) (B) in dichloromethane-*d*_2_.

In the test with complex **a**, the type **3** cluster is produced in a minor amount, and it is consequently possible to isolate the type **2** cluster **2a** by recrystallization followed by column chromatography. ^31^P NMR analysis of the cluster apparently confirms the result of mass spectrometry: after the purification, [Au_13_(di-NHC^a^)_2_(PPh_3_)_4_Cl_4_]^+^**2a** shows four quartets with equal integration in its ^31^P spectrum, as reported in Figure 4. Conversely, in the experiment that involves complex **b**, the type **3** cluster **3b** is present in greater amount compared to type **2** cluster **2b** and is easier to isolate. This cluster has been characterized by ^31^P NMR as well and shows a singlet at 60.83 ppm. With this information at hand, it is also possible to interpret the ^31^P NMR spectrum of the original **2b**/**3b** cluster mixture (Figure 4) and to attribute the signals stemming from the **2b** cluster. ^31^P NMR spectra of **2b** are characterized by four quartets with the same integration, similar to what was found with the **2a** cluster. Thus, type **2** clusters appear to feature a lower degree of symmetry in solution, with all phosphine ligands being magnetically inequivalent (see however below), whereas the arrangement of the phosphine ligands is more symmetrical in the case of the **3b** cluster.

**Figure 5:**
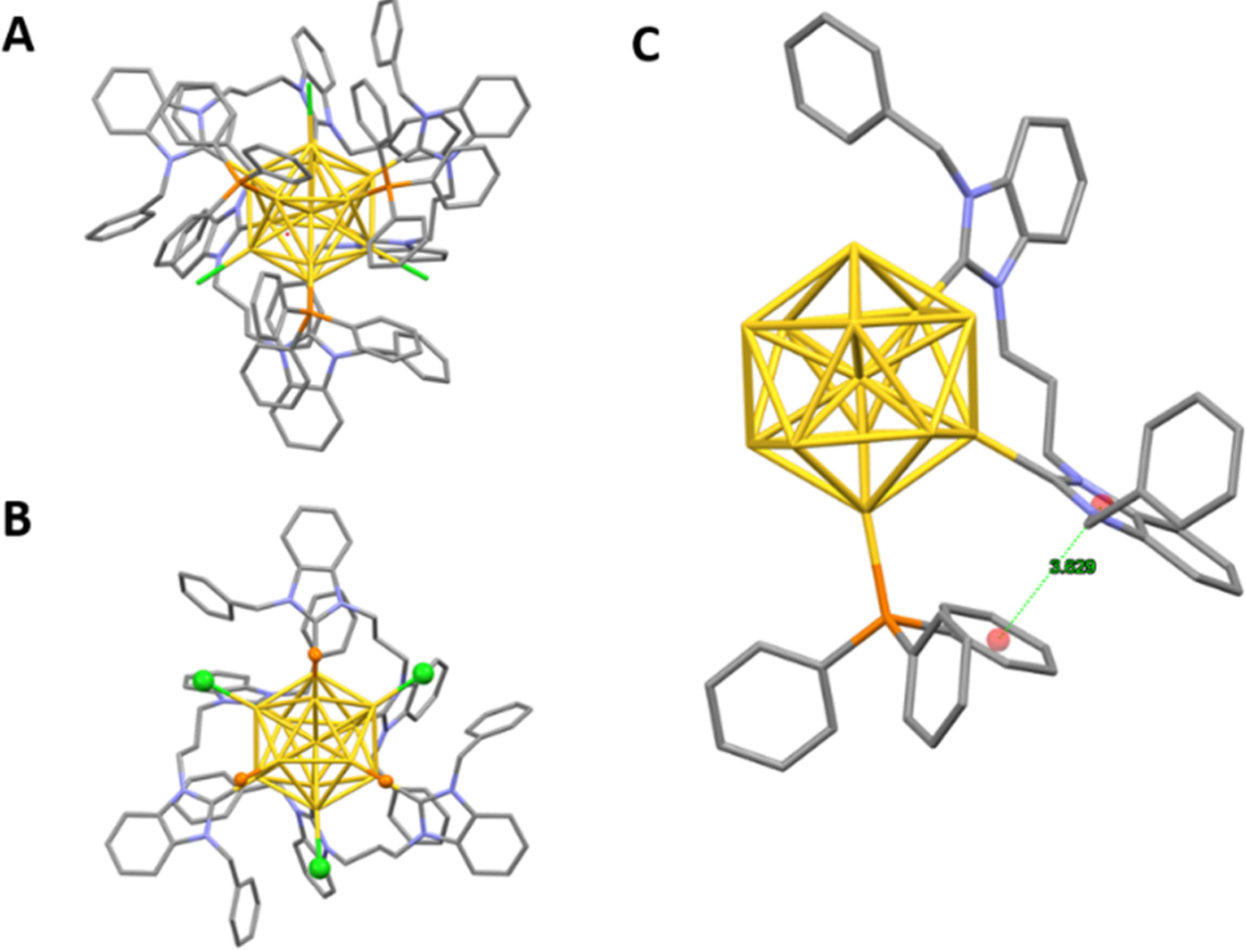
X-ray structure of **3b**. Full view of the cluster structure (A), full view after removal of the PPh_3_ phenyl groups (B), and π–π interaction among NHCs and PPh_3_ ligands (C). Gold atoms are highlighted in yellow, chloride anions in green, phosphorus in orange, nitrogen in purple, and carbon in gray. For clarity, hydrogens are omitted in all figures.

We were able to obtain single crystals of cluster **3b** suitable for X-ray diffraction analysis, layering n-hexane on a dichloromethane solution. The molecular structure of **3b** is reported in Figure 5.

The cluster core presents the well-known Au_13_ icosahedral structure. The PPh_3_ ligands, highlighted in orange in Figure 5, are all terminally bonded to three gold atoms which define a triangular face of the metallic core. Taking this face as reference (Figure 5B), chloride ions are staggered at about 60° from PPh_3_ positions and bonded to Au atoms, which compose the other three triangular faces, sharing one side with the reference face. The remaining six Au atoms bind to three di-NHC ligands, which form a helical system with a *C*3 symmetry. This arrangement of the di-NHC ligands renders the cluster structure chiral. The structure is fully in agreement with the ^31^P NMR spectrum and Q-TOF analysis. Interestingly, π–π-interactions are present only between NHC-imidazole rings and PPh_3_-phenyl groups, where the average centroid distance is equal to 3.629 Å (Figure 5C). The Au–Au distances have a value between 2.888 and 3.100 Å. To conclude, the average value of Au–Cl, Au–P, and Au–C bonds are, respectively, 2.367, 2.295, and 2.049 Å, in agreement with data reported in the literature. The positive charge of the dicationic cluster is neutralized by a chloride and a disordered OH^–^ anion. Several dichloromethane molecules are also present in the crystal packing of the compound.

We tried to further promote the synthesis of the various clusters by prolonging the heating time. If the reaction mixture with complex **c** is kept at 40 °C for 3 days, cluster **1c** is produced as the main product, although it is still possible to record the presence of the reagent cluster and of other clusters around 2000–2300 *m*/*z* by Q-TOF analysis. Even longer heating times increase the complexity of the resulting product mixture, affording clusters **2c** and **3c** only in detectable amounts in Q-TOF spectra; hence, the reaction was stopped after 3 days and **1c** was purified. In this case, both ^1^H and ^31^P NMR can be employed to characterize the AuNC and compare it with the starting [Au_11_(PPh_3_)_8_Cl_2_]Cl. The ^1^H NMR spectrum of **1c** in dichloromethane-*d*_2_ (see Figure S15 in the SI) shows shifted aromatic signals compared with the pure phosphine cluster. Furthermore, it is possible to identify signals from the di-NHC ligand, in particular from the propylene bridge and the isopropyl wingtip substituents, that were very broad and featureless in the case of the other clusters isolated in this work. In the ^31^P NMR spectrum instead, the singlet exhibited by [Au_11_(PPh_3_)_8_Cl_2_]Cl at 52.27 ppm is split into two broad signals, centered at 52.70 ppm (5P) and 51.36 ppm (1P), respectively. Thus, NMR analysis confirms the coordination of one di-NHC ligand to the Au_11_ core, with the production of a cluster having [Au_11_(di-NHC^c^)(PPh_3_)_6_Cl_2_]^+^ stoichiometry.

Experiments with prolonged heating at 40 °C were performed also with complexes **a** and **b**, aiming in particular at accelerating the reaction rate and at selectively producing type **3** clusters; for this reason, a 1:3 [Au_11_]/[(di-NHC)Au_2_Cl_2_] molar ratio was also employed. However, in the case of the starting complex **a**, use of three equivalents of complex caused the formation of a white precipitate in the reaction solution that was visible already after 24 h. Using Q-TOF and ^1^H NMR, the precipitate was found to contain the dicationic complex [Au_2_(di-NHC^a^)_2_]^2+^, which is produced through degradation of **a** and is apparently inert toward reaction (both metathesis and addition) with the clusters. Its low reactivity can be justified with its low solubility in dichloromethane, high positive charge (causing electrostatic repulsion with the positively charged clusters), and high stability, arising from its metallacyclic structure. Using instead **b** as a reagent, within 5 days at 40 °C, the only cluster present in solution is **3b**, which can be conveniently purified by column chromatography. Furthermore, if the reaction is stopped after 3 days, both **2b** and **3b** clusters can be isolated. The ^31^P NMR spectrum of **3b** prepared in this way presents one singlet at 60.83 ppm, as in the case of the sample prepared through the previously described methodology. Surprisingly, the ^31^P NMR spectrum referred to **2b** derived from this synthesis is instead different, despite exhibiting exactly the same Q-TOF peak: although previously synthesized **2b** presents four quartets (Figure 4), the same cluster prepared with the last procedure (i.e., upon prolonged heating at 40 °C) and hereafter termed **2b′**, presents only one singlet, centered at 27.42 ppm. This suggests that the cluster with stoichiometry [Au_13_(di-NHC^b^)_2_(PPh_3_)_4_Cl_4_]^+^ can exist in at least two different isomeric forms, and if it is assumed that both isomers share the same icosahedral geometry of the Au_13_ core (which appears plausible), then the two isomeric forms are geometrical isomers featuring different degrees of symmetry. The existence of AuNCs in two isomeric forms is rare but has been reported previously.^55,63,64^ We are currently seeking to structurally characterize these two isomers and to establish whether they are formed independently or convert one into the other.

**Figure 6:**
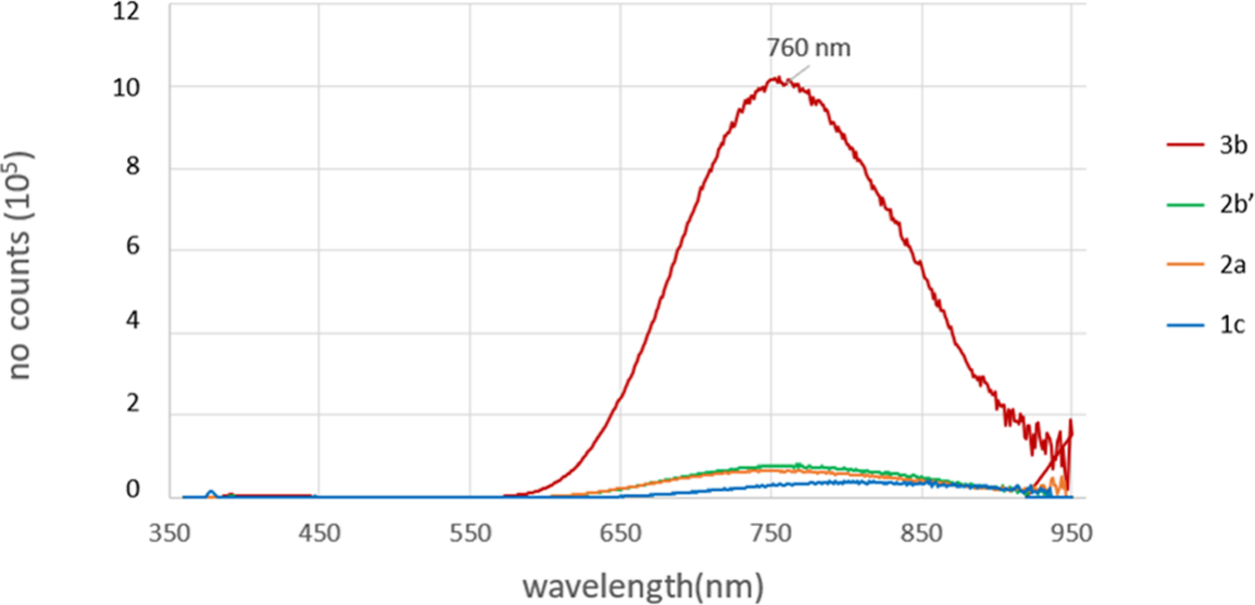
Emission spectra of **3b** (red), **2b′** (green), **2a** (orange), and **1c** (blue) in dichloromethane. All spectra have been normalized with the related cluster absorbance at 350 nm.

The absorption properties of AuNCs were evaluated with UV–vis spectroscopy (see Figure S33 in the SI). **2a**, **2b′** and **3b** clusters all show a weaker absorption band at around 430 nm and a much stronger one with a maximum placed around 340–350 nm. Cluster **1c** behaves instead differently, exhibiting two bands at 419 and 315 nm. The luminescence properties of the AuNCs were also studied by exciting all of the samples at 350 nm. The four emission spectra are reported in Figure 6, normalized for the cluster absorbance values at the excitation frequency.

All AuNCs present a broad emission band centered at 760 nm, yet **3b** shows by far the most intense emission, with a 44% quantum yield that ranks among the highest ever reported for a superatomic Au_13_ or larger gold cluster.^65,66^ This peculiar optical property of **3b** can be rationalized considering its low-symmetry degree (i.e., only a *C*_3_ axis is present in the structure) and its rather high rigidity due to the presence of three chelating di-NHC ligands, which strongly affect the emission quantum yield of the compound.^35^

Finally, to shed more light on the reaction sequence leading to type **2** and type **3** clusters, we decided to monitor the reaction of an isolated sample of cluster **1b** with 1 equiv of complex **b** at 40 °C. The reaction produced as expected both clusters **2b** and **3b**, in line with the original reaction starting from the parent phosphine cluster reagent. This is a confirmation of the stepwise nature of the process. However, when we performed a control experiment using only the starting **1b** cluster, without addition of complex **b**, we found that the system was still able to evolve producing only **2b** as cluster product, without the formation of cluster **3b**. Consequently, we have to conclude that the reaction step leading from type **1** to type **2** clusters must not be necessarily viewed as a complex addition reaction but can take place upon thermal rearrangement of type **1** clusters, albeit this rearrangement might still involve etching of a di-NHC gold(I) complex from a type **1** cluster and its subsequent addition to another type **1** cluster. More work is needed to ascertain which is the preferred mechanistic option in this reaction step. The experiments also confirm that the reaction step leading from type **2** to type **3** clusters is a ligand metathesis process that necessitates the presence of a di-NHC gold(I) complex.

## Conclusions

We have reported a new method to synthesize AuNCs stabilized by a mixed ligand sphere composed of PPh_3_ and di-NHC ligands. The method is based on the reaction of a preformed, PPh_3_-stabilized AuNC with dinuclear di-NHC gold(I) complexes and enables the production and isolation of clusters with different nuclearity and ligand stoichiometry, depending on the di-NHC properties and reaction conditions. We are currently investigating the extension of this methodology to other starting phosphine-stabilized AuNCs and di-NHC complexes of gold(I), as well as to complexes of other metals, that could enable faster metathesis (e.g., with silver(I) complexes) and/or allow to prepare heterobimetallic clusters. A detailed reaction monitoring that has been conducted with Q-TOF high-resolution mass spectrometry allows us to hypothesize a reaction sequence involving a first metathesis reaction to produce type **1** clusters, followed by a cluster rearrangement/di-NHC gold complex addition to obtain type **2** clusters and a further metathesis that provides type **3** clusters. Theoretical mechanistic studies are planned to investigate in greater detail all of these reaction steps. Finally, the obtained AuNCs have been characterized with different techniques, including ^1^H and ^31^P NMR spectroscopy, UV–vis and emission spectroscopy, and X-ray single-crystal diffraction. These studies have provided evidence that at least one of the clusters produced in this work can exist in two isomeric forms (i.e., cluster **2b**/**2b′**). Furthermore, a strong luminescence has been recorded for cluster **3b**, which is in line with the low degree of symmetry and high rigidity exhibited by this cluster. Evidence of such property calls for more detailed studies involving the evaluation of quantum yields and decay times, as well as on the effect of different ligands on the emission properties, which will be conducted in due course.

## Supporting Information Available

Synthetic procedures and complete characterization data for starting compounds and cluster products (PDF)
