# Supplementary material for: From Au11 to Au13: Tailored Synthesis of Superatomic Di-NHC/PPh3-Stabilized Molecular Gold Nanoclusters
Source: Inorg Chem. 2023 Jan 13;62(4):1383–93. doi: 10.1021/acs.inorgchem.2c03331 (PMC9890486; doi:10.1021/acs.inorgchem.2c03331)
Supplement: Supplementary file 1 — ic2c03331_si_001.pdf [file ic2c03331_si_001.pdf]

Electronic supporting information

From Au_{11} to Au_{13}: tailored synthesis of superatomic di-NHC/PPh_3 stabilized molecular gold nanoclusters

Matteo Bevilacqua†, Marco Roverso†, Sara Bogialli†, Claudia Graiff# and Andrea Biffis*†

† Dipartimento di Scienze Chimiche, Università degli Studi di Padova, Via F. Marzolo 1, 35131 Padova, Italy. Email andrea.biffis@unipd.it

Dipartimento di Scienze Chimiche, della Vita e della Sostenibilità Ambientale, Università degli Studi di Parma, Parco Area delle Scienze 17/A, 43124 Parma, Italy

Table of contents

1.0 Characterization data 2

1.1 NMR spectra 2

1.1.1 ^{1}H NMR spectra of di-imidazolium salts 2

1.1.2 ^{1}H NMR spectra of di-NHC complexes 4

1.1.3 ^{1}H and ^{31}P NMR spectra of NHC-PPh_3 protected AuNCs 6

1.2 Q-TOF HRMS analyses 10

1.2.1 Q-TOF HRMS analyses of NHC-PPh_3 AuNCs 10

1.3 Reaction monitoring by HRMS 15

1.3.1 Q-TOF HRMS analyses of experiment involving **a** 18

1.3.2 Q-TOF HRMS analyses of experiment involving **b** 18

1.3.3 Q-TOF HRMS analyses of experiment involving **c** 18

1.4 Emission spectra and QY value of NHC-PPh_3 AuNCs 19

1.5 Emission spectra and QY value of NHC-PPh_3 AuNCs 20

1.6 X ray data collection for compound **3b** 21

2.0 Bibliography 22

1.0 Characterization data

1.1 NMR spectra

1.1.1 ^{1}H NMR spectra of di-imidazolium salts

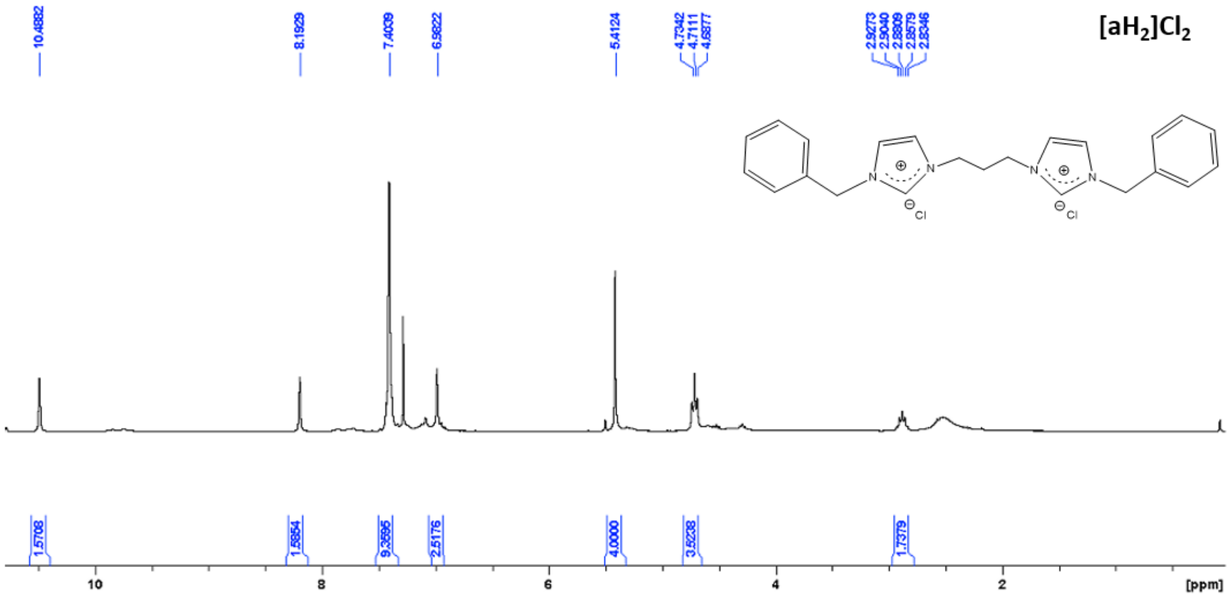

Figure S1: ^{1}H NMR spectra of [**a**H$_2$]Cl$_2$ in $CDCl_3$.

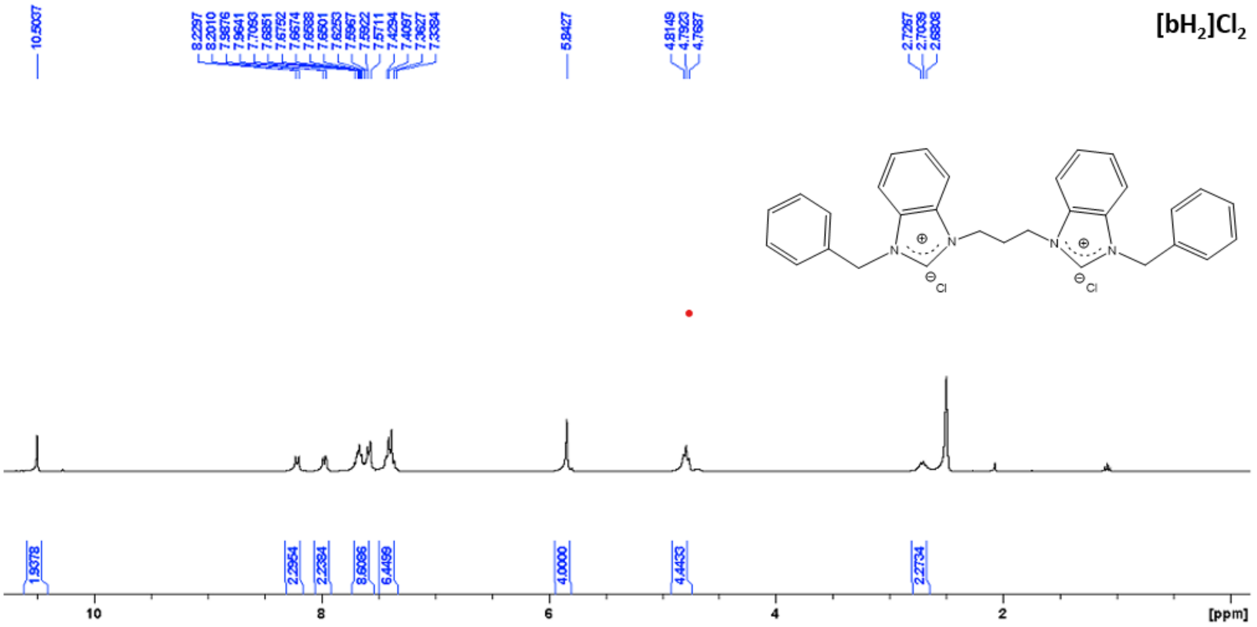

Figure S2: ^{1}H NMR spectra of [**b**H$_2$]Cl$_2$ in DMSO-d^6.

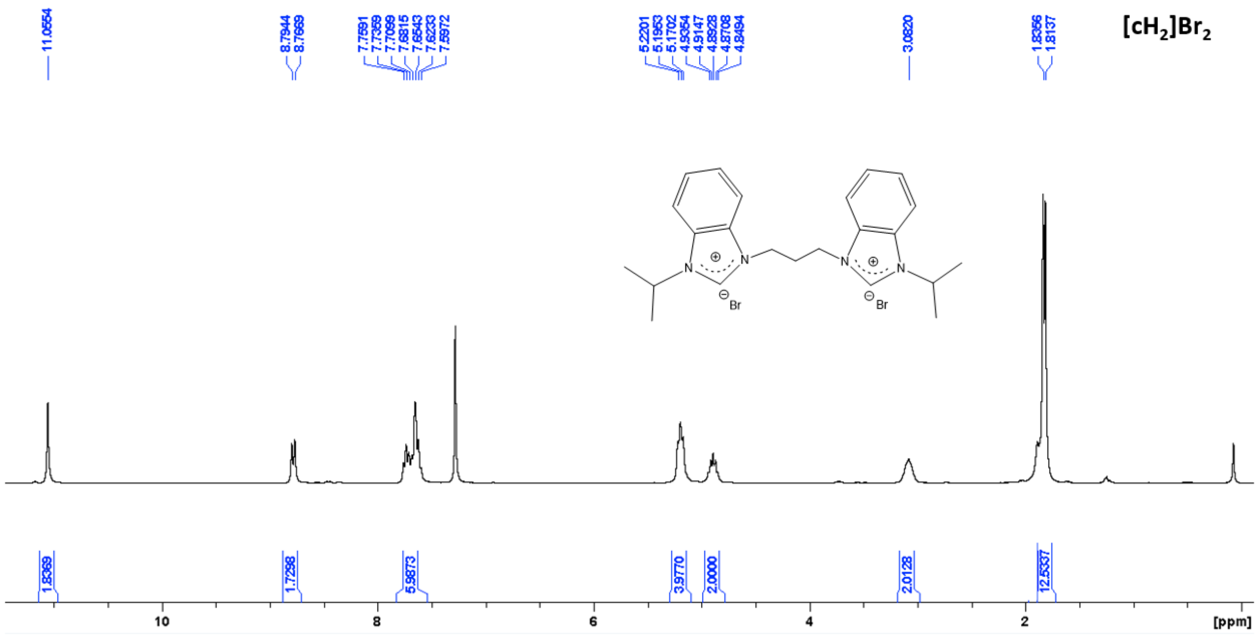

Figure S3: ^{1}H NMR spectra of [**c**H$_2$]Br$_2$ in CDCl$_3$.

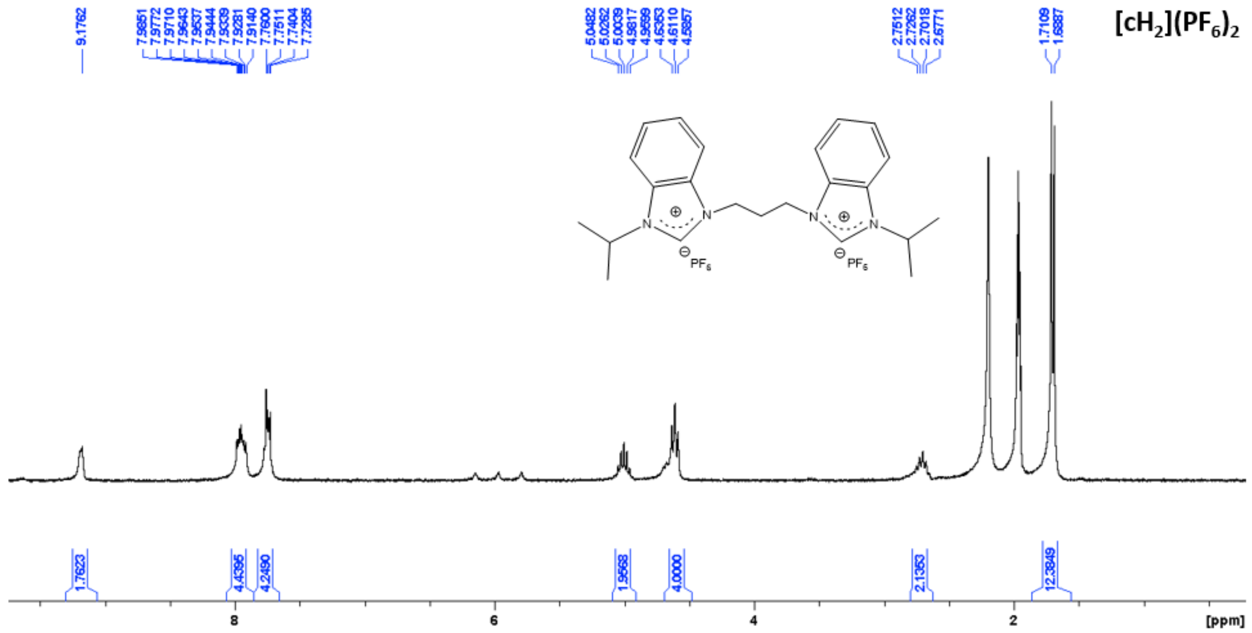

Figure S4: ^{1}H NMR spectra of [**c**H$_2$](PF$_6$)$_2$ in CD$_3$CN.

1.1.2 ^{1}H NMR spectra of di-NHC complexes

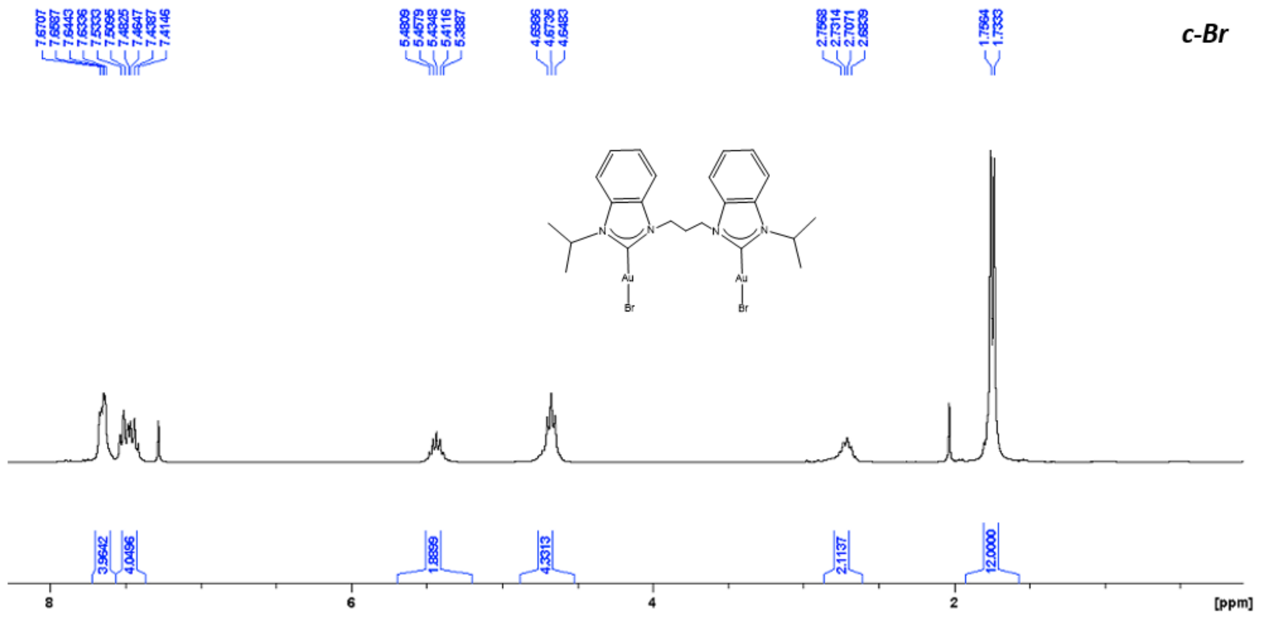

Figure S5: ^{1}H NMR spectra of **c-Br** in $CDCl_3$.

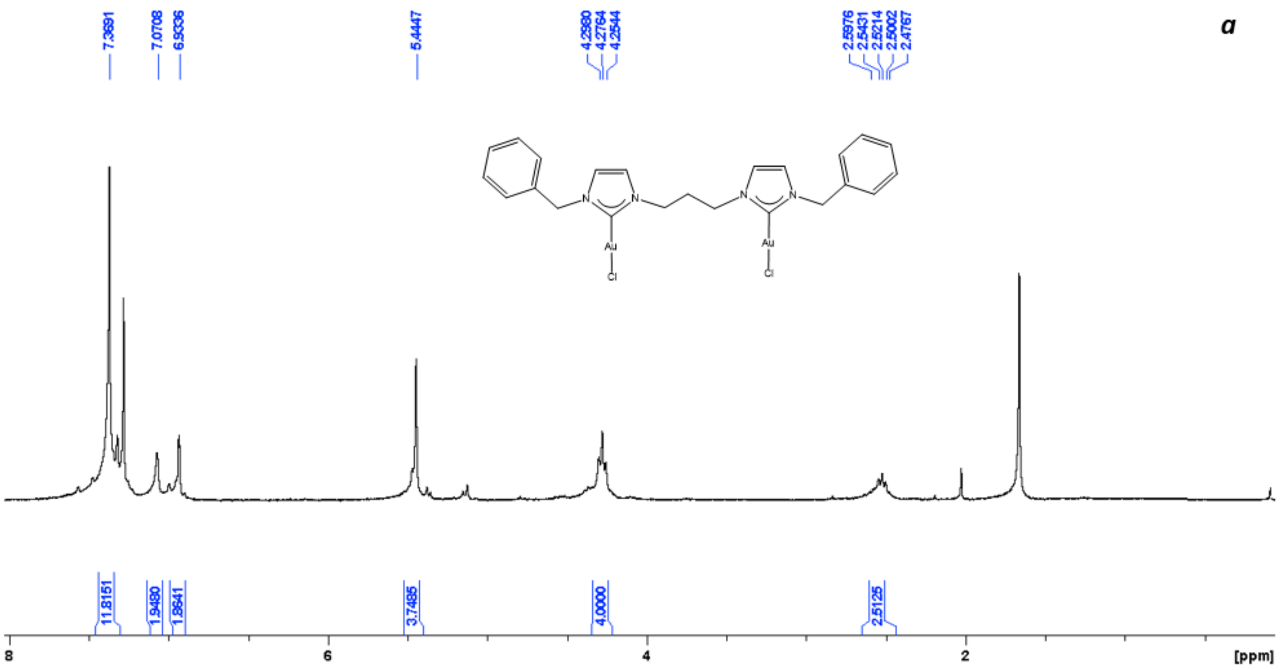

Figure S6: ^{1}H NMR spectra of **a** in $CDCl_3$.

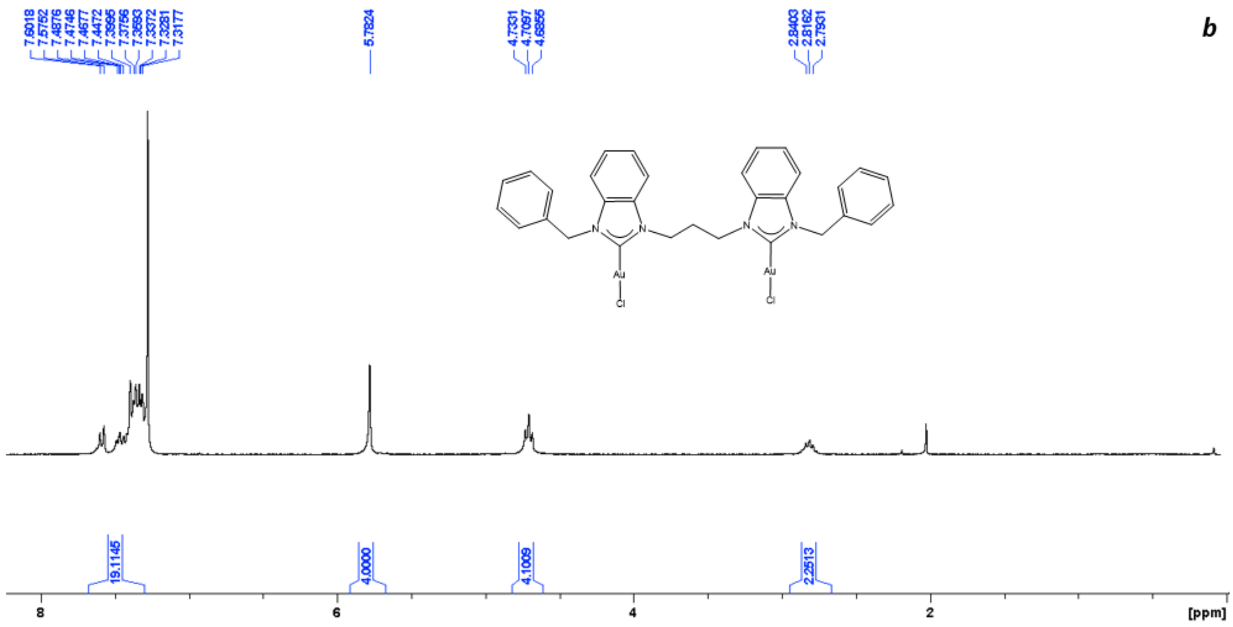

Figure S7: ^{1}H NMR spectra of **b** in $CDCl_3$.

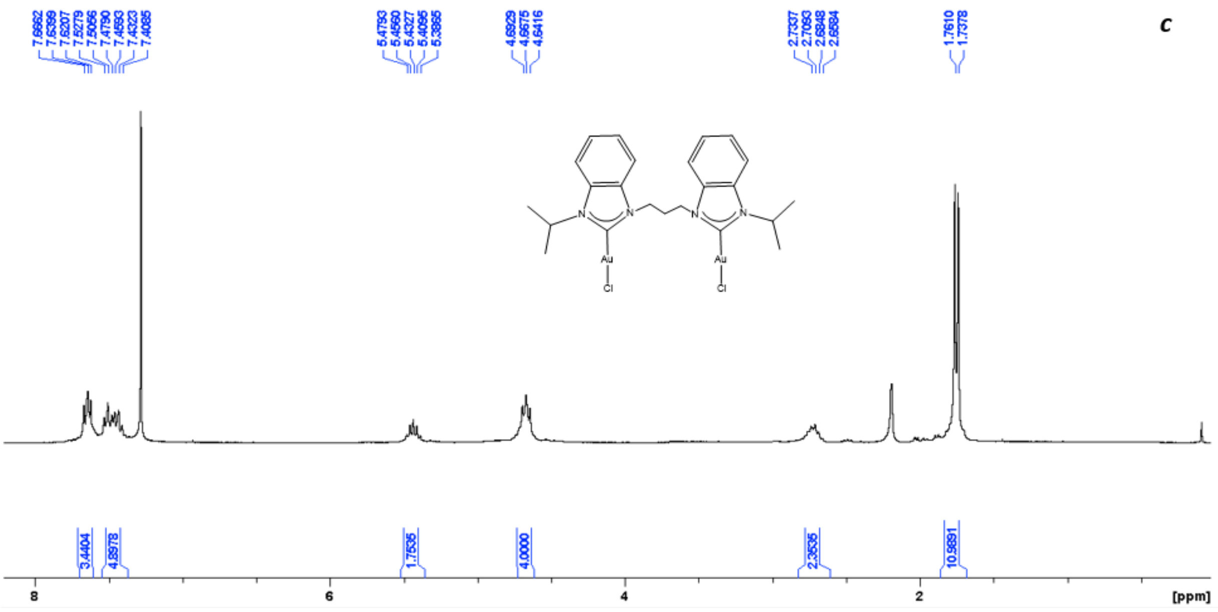

Figure S8: ^{1}H NMR spectra of **c** on $CDCl_3$.

1.1.3 ^{1}H and ^{31}P NMR spectra of NHC-PPh$_3$ protected AuNCs

The ^{1}H NMR spectra of **2a**, **2b, 2b'** and **3b** are not reported since they are not interpretable. For demonstration, the protonic spectrum of **2a** in DCM-d^2 is reported.

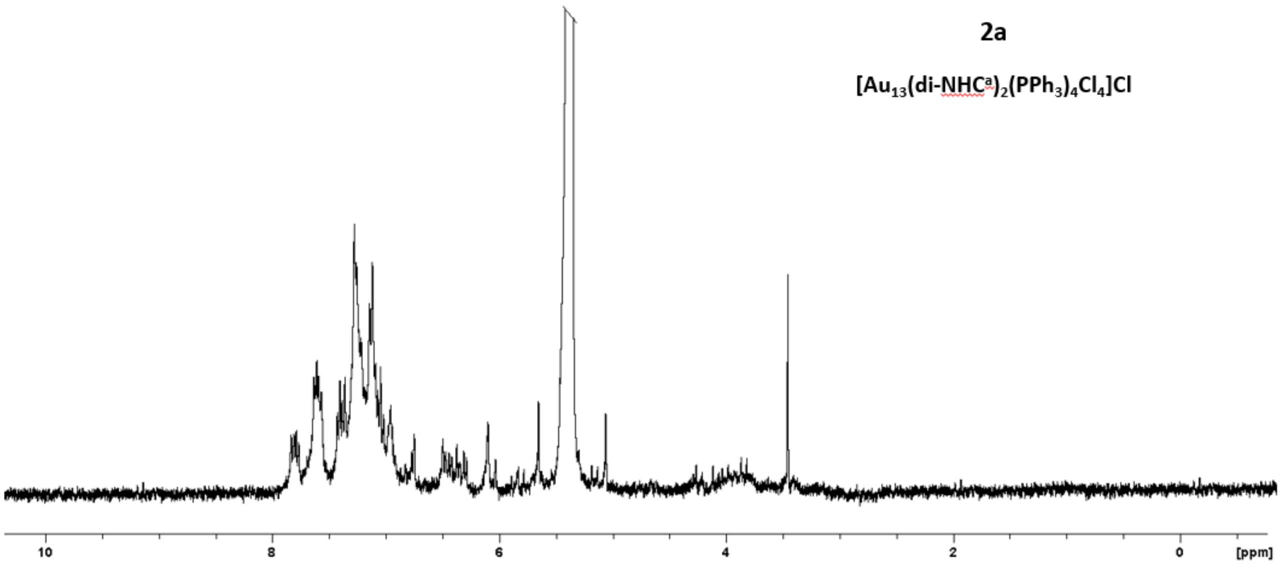

Figure S9: ^{1}H NMR spectra of **2a** in CD_2Cl_2.

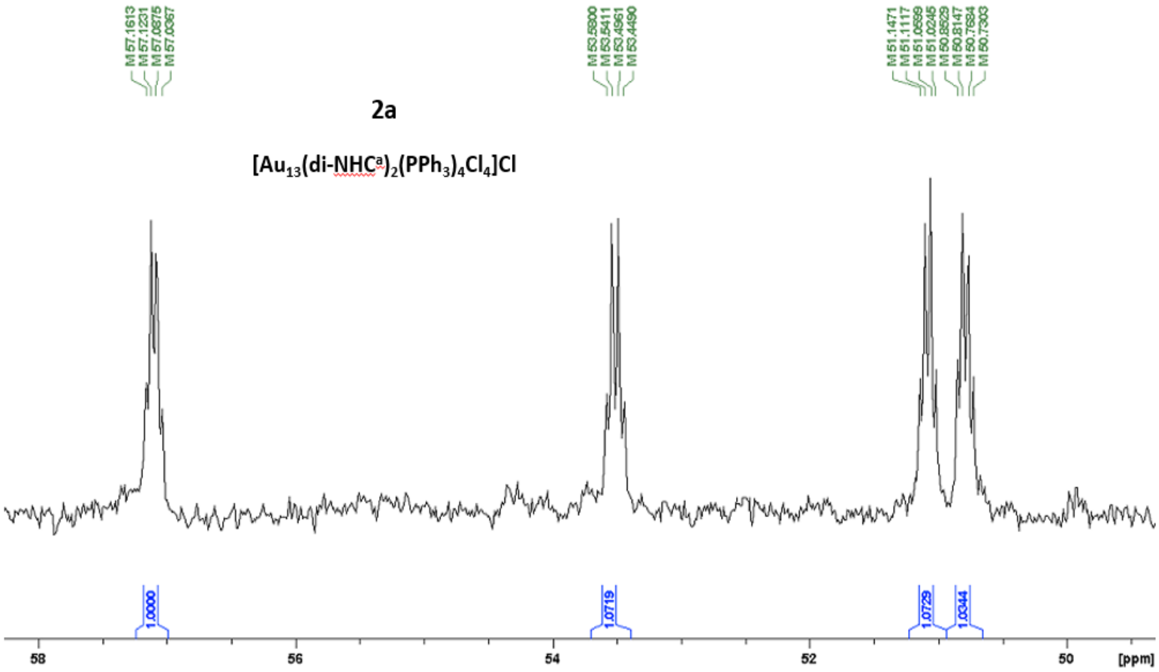

Figure S10: ^{31}P NMR spectra of **2a** in CD_2Cl_2.

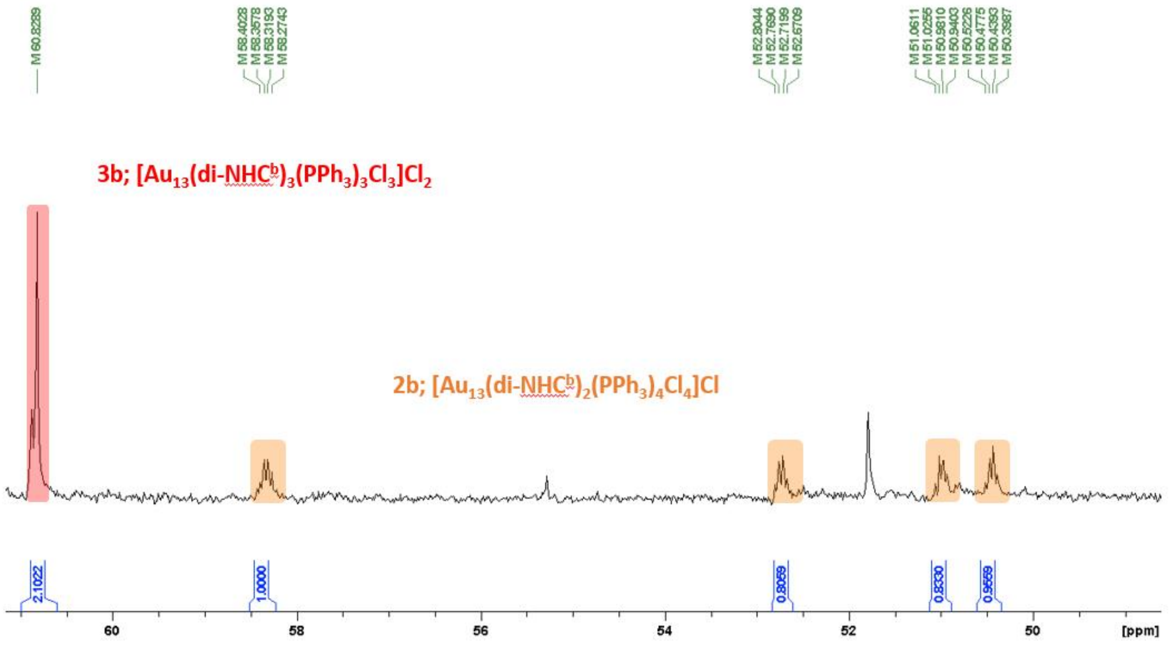

Figure S11: ^{31}P NMR spectra of reaction mixture dissolved in CD_2Cl_2 containing **2b** (orange) and **3b** (red) before chromatographic purification.

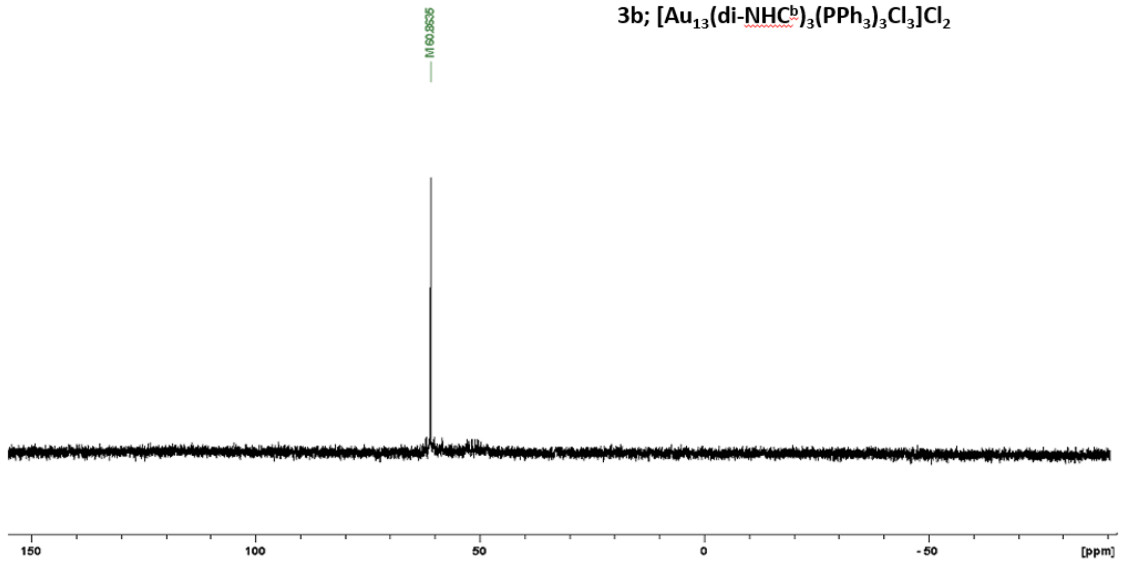

Figure S12: ^{31}P NMR spectra of **3b** in CD_2Cl_2 after chromatographic purification.

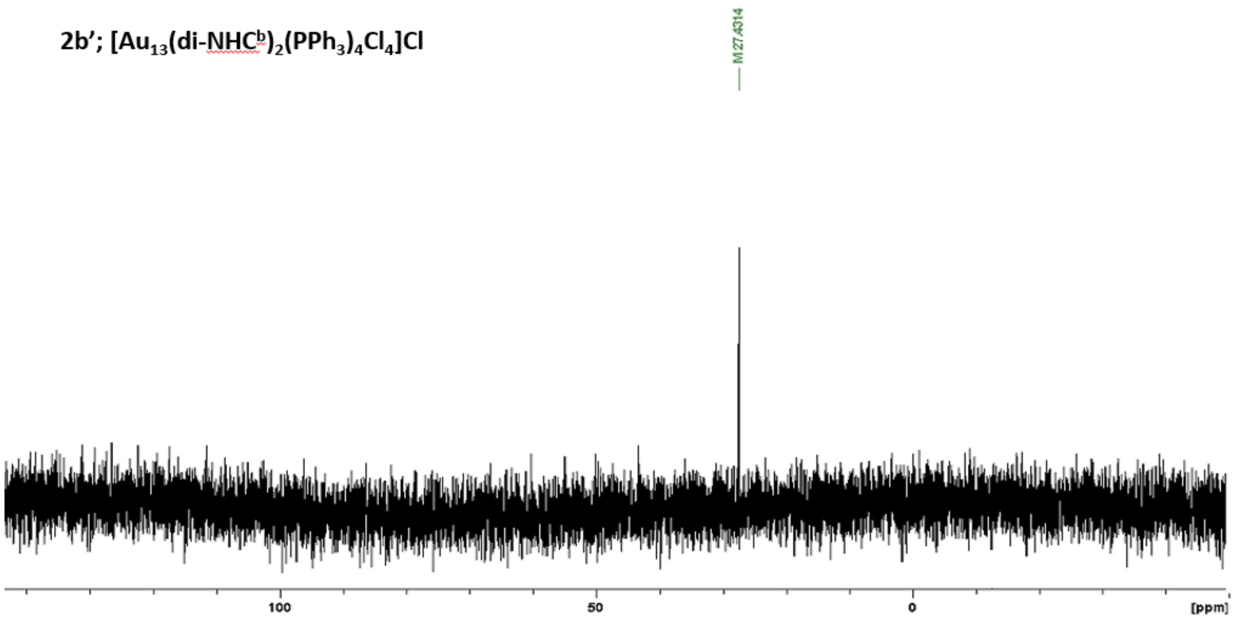

Figure S13: ^{31}P NMR spectra of **2b'** in CD_2Cl_2.

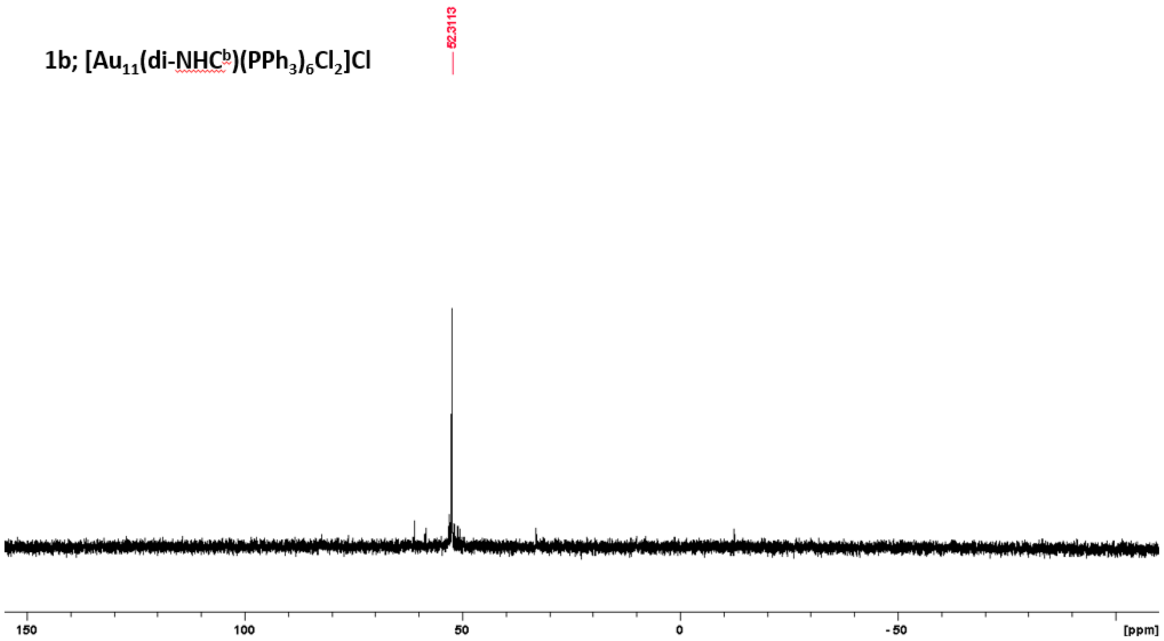

Figure S14: ^{31}P NMR spectra of **1b** in CD_2Cl_2.

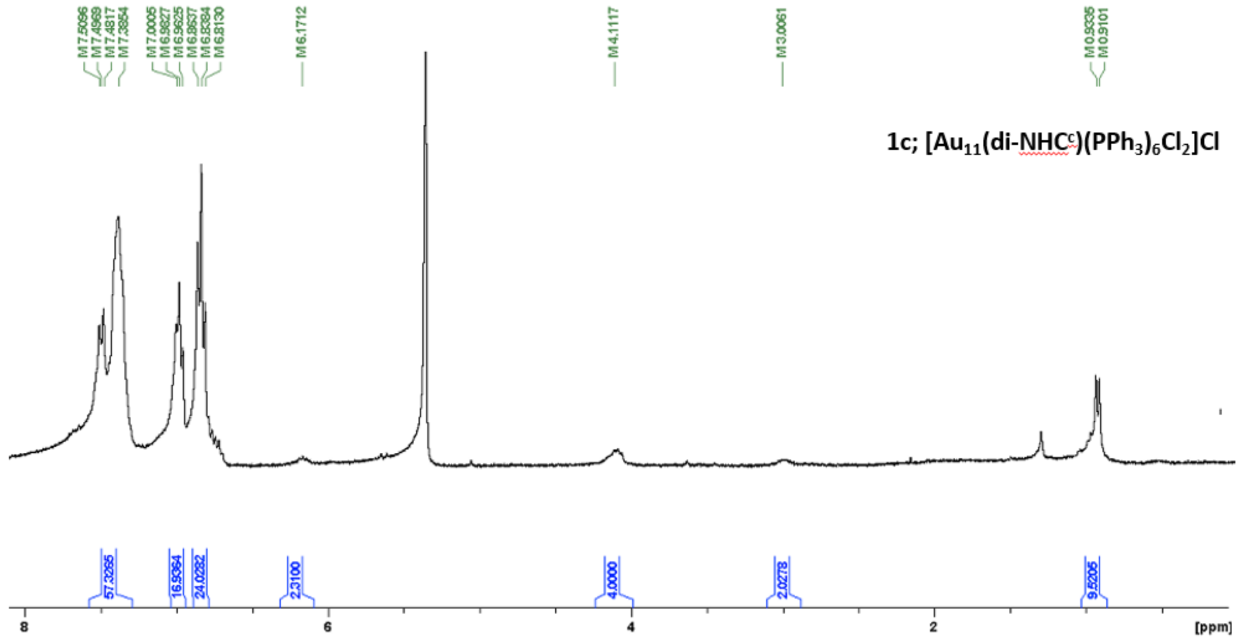

Figure S15: ^{1}H NMR spectra of **1c** in CD_2Cl_2.

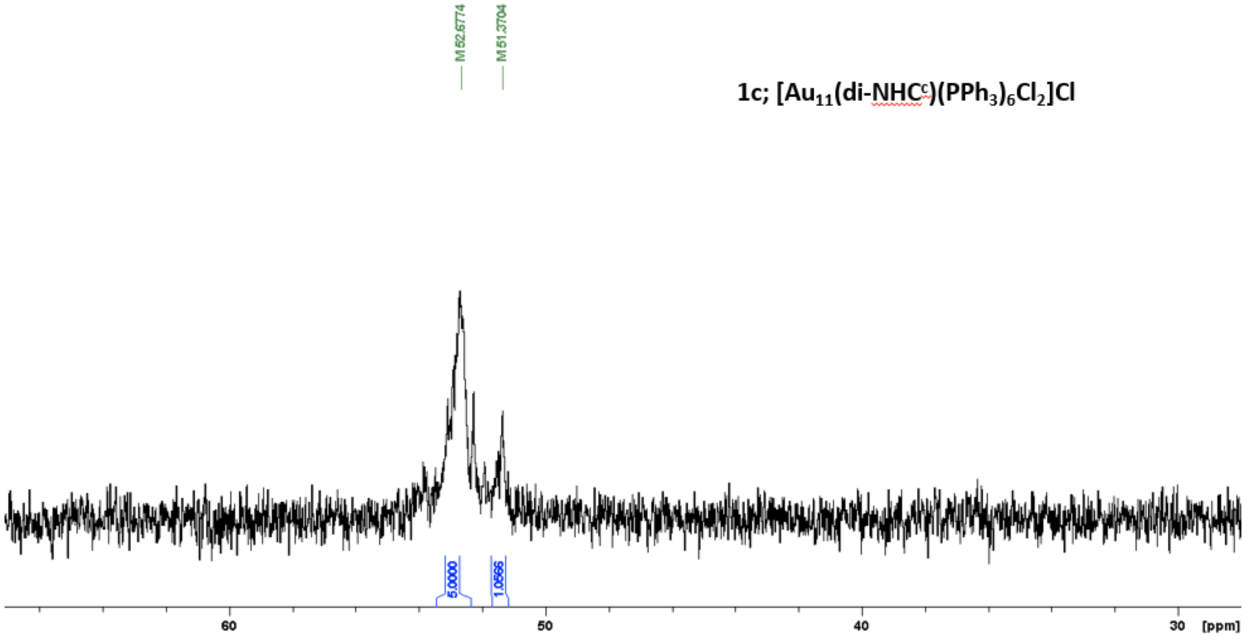

Figure S16: ^{31}P NMR of **1c** in CD_2Cl_2.

1.2 Q-TOF HRMS analyses

1.2.1 Q-TOF HRMS analyses of NHC-PPh3 AuNCs

In all Q-TOF HRMS analyses a fragmentation peak centred at 721 m/z is present, referred to the $[Au(PPh_3)_2]^+$ fragment. This cationic complex is not present in all reported ^{31}P NMR spectra.

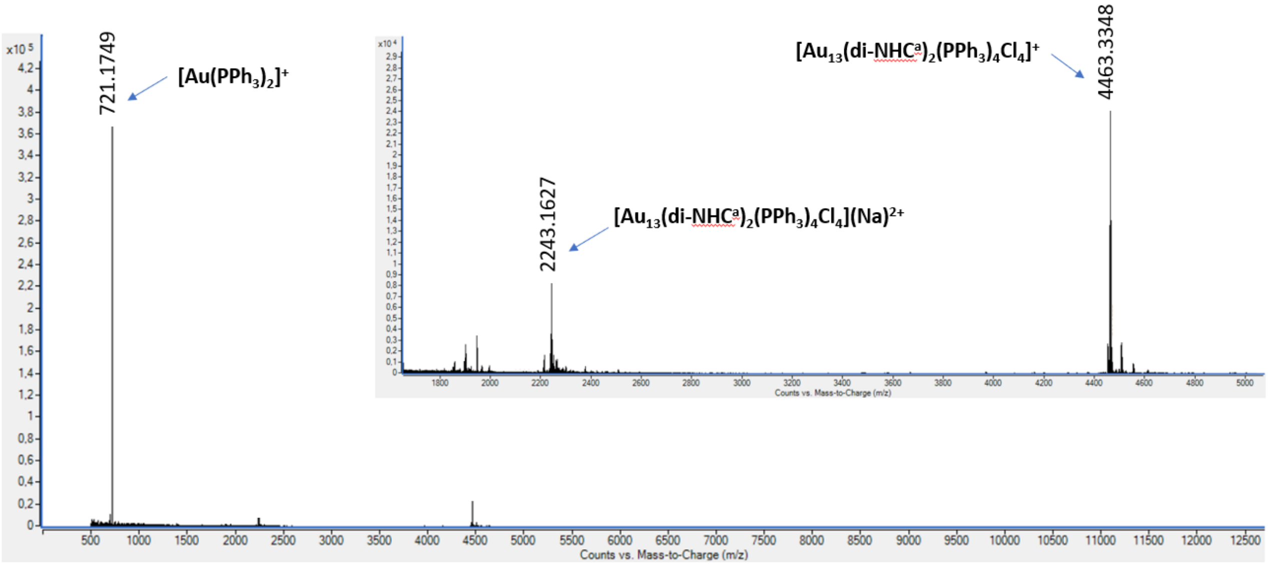

Figure S17: Q-TOF analysis of **2a** in CH_2Cl_2.

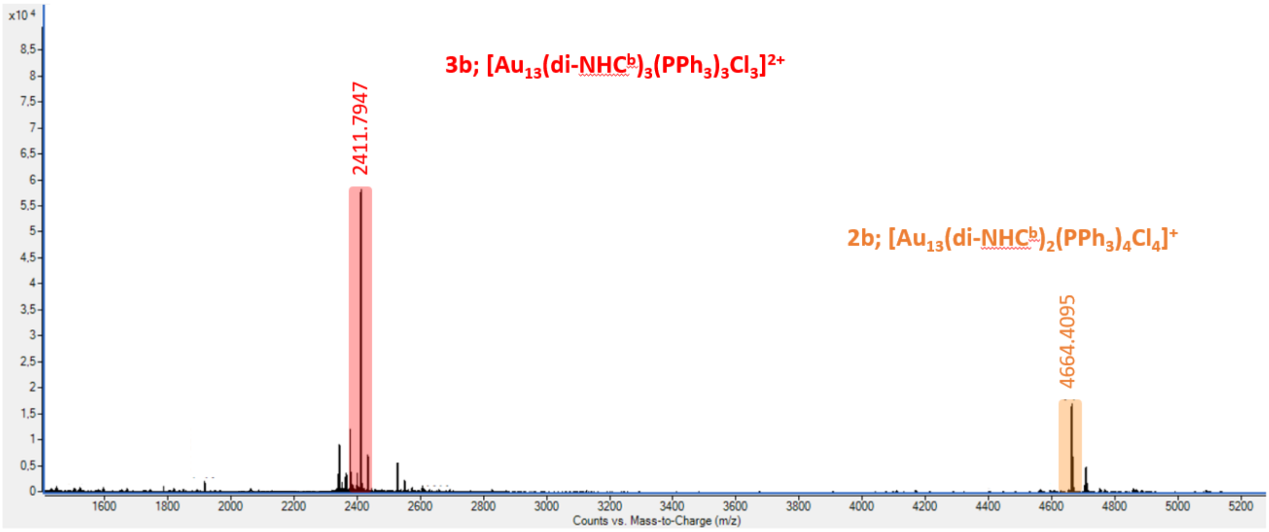

Figure S18: Q-TOF analysis of reaction mixture containing **2b** and **3b** before purification provided by chromatographic column.

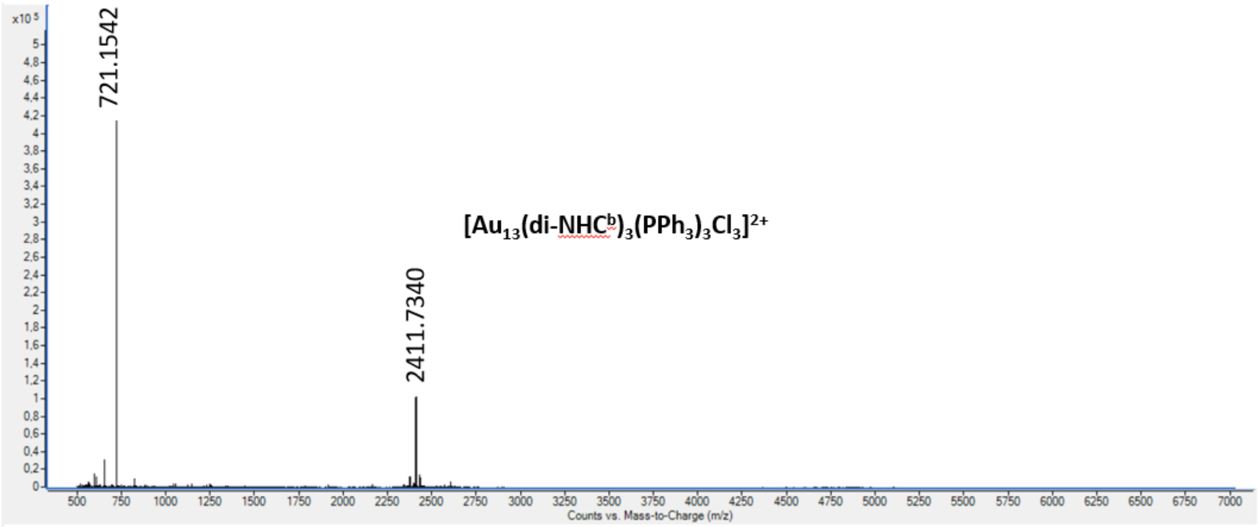

Figure S19: Q-TOF analysis of **3b** in CH_2Cl_2.

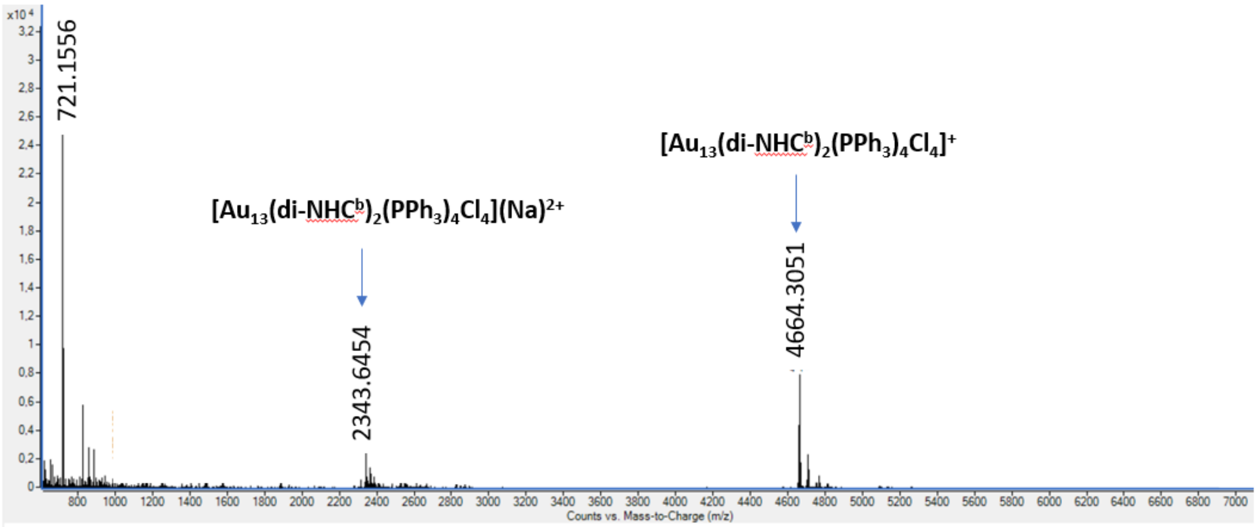

Figure S20: Q-TOF analysis of **2b’** in CH_2Cl_2.

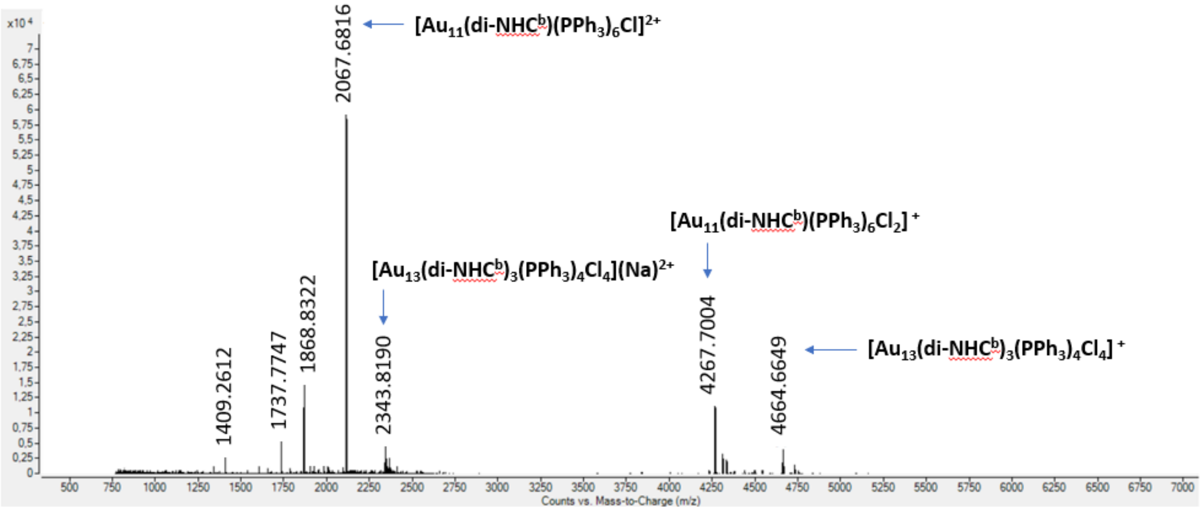

Figure S21: Q-TOF analysis of **1b** in CH_2Cl_2.

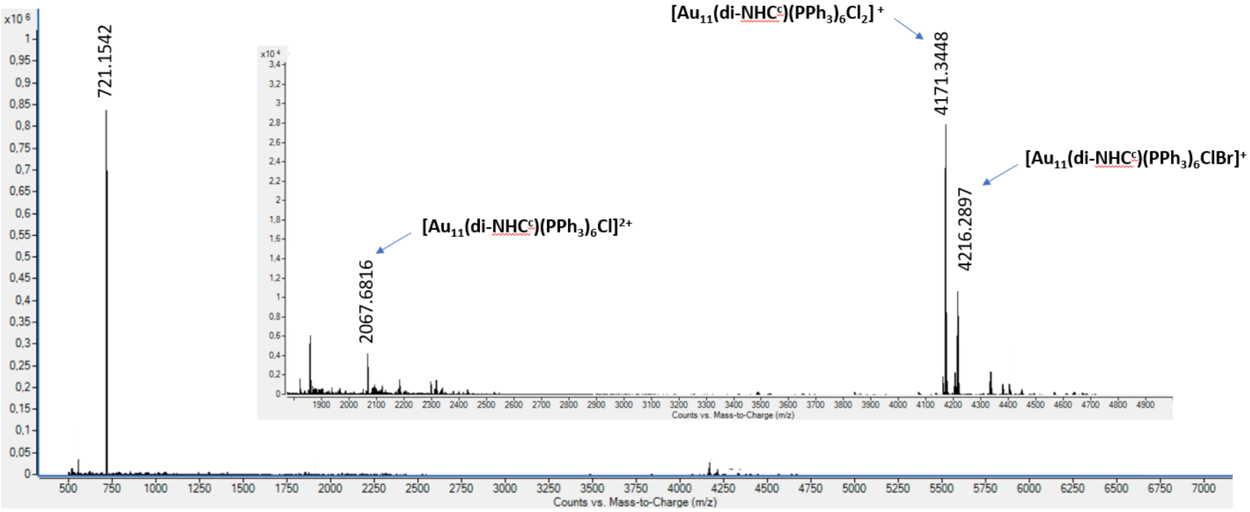

Figure S22: Q-TOF analysis of **1c** in CH_2Cl_2.

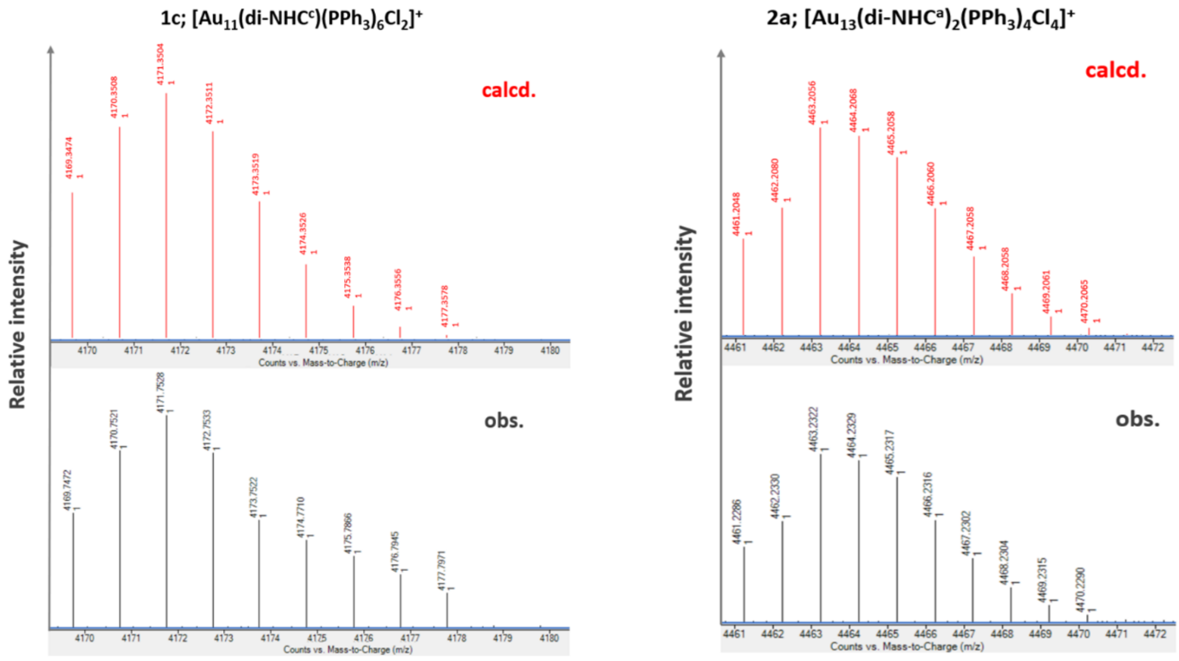

Figure S23: measured (black) and calculated (red) Q-TOF spectrum of **1c** and **2a**.

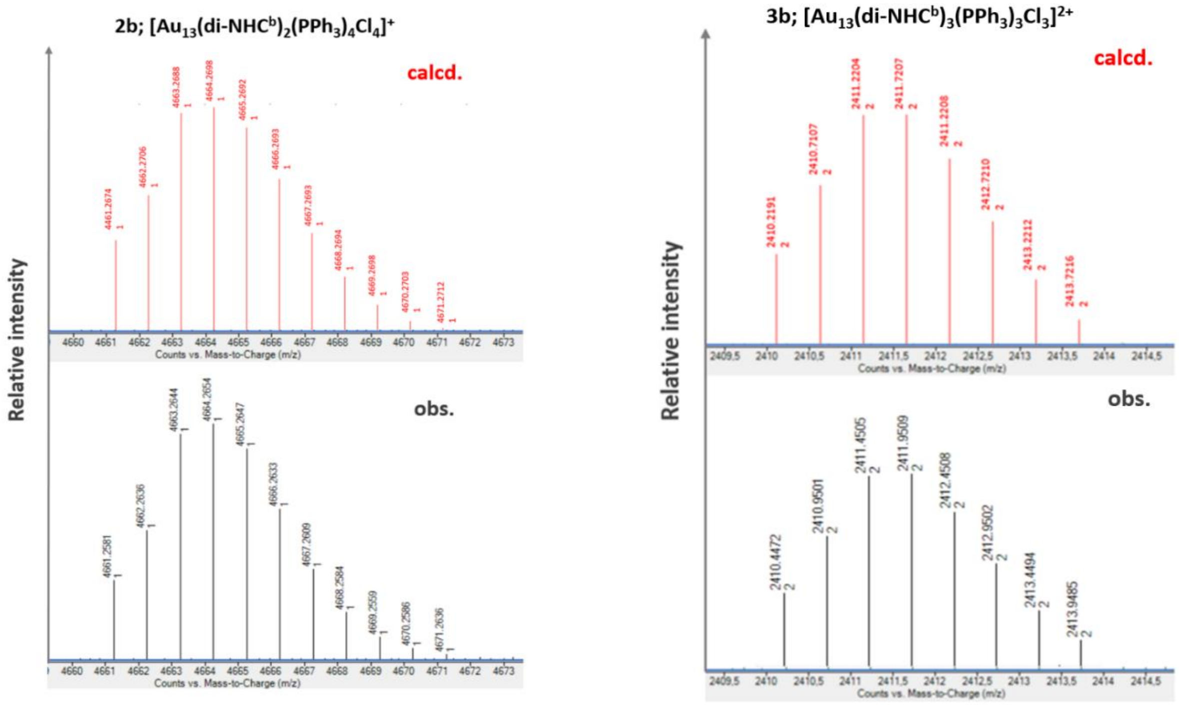

Figure S24: measured (black) and calculated (red) Q-TOF spectrum of **2b** and **3b**.

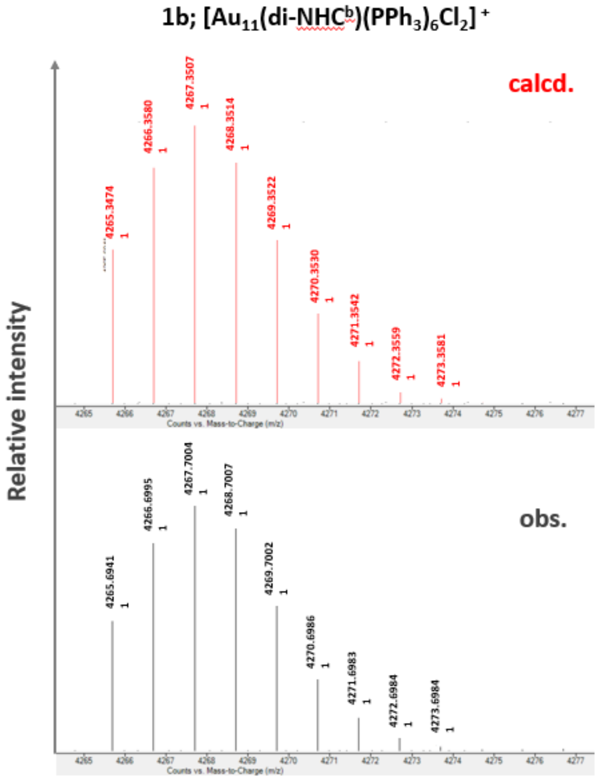

Figure S25: measured (black) and calculated (red) Q-TOF spectrum of **1b**.

Cluster name	measured (m/z)	calculated (m/z)	Δm(ppm)	formula
**2a**	4461.2188	4461.2048	3.1381	$[Au_{13}(C_{23}H_{24}N_4)_2(PC_{18}H_{15})_4Cl_4]^+$
	4505.2070	4505.1543	11.698	$[Au_{13}(C_{23}H_{24}N_4)_2(PC_{18}H_{15})_4BrCl_3]^+$
	2242.1688	2242.0970	32.023	$[Au_{13}(C_{23}H_{24}N_4)_2(PC_{18}H_{15})_4Cl_4](Na)^{2+}$
**1b**	4265.6941	4265.3474	81.276	$[Au_{11}(C_{31}H_{28}N_4)(PC_{18}H_{15})_6Cl_2]^+$
	2115.3641	2115.1890	82.775	$[Au_{11}(C_{31}H_{28}N_4)(PC_{18}H_{15})_4Cl]^{2+}$
**2b**	2342.1240	2342.1283	-1.8359	$[Au_{13}(C_{31}H_{28}N_4)_2(PC_{18}H_{15})_4Cl_4](Na)^{2+}$
	4705.1975	4705.2169	-4.1230	$[Au_{13}(C_{31}H_{28}N_4)_2(PC_{18}H_{15})_4BrCl_3]^+$
	2342.1240	2342.1283	-1.8359	$[Au_{13}(C_{31}H_{28}N_4)_2(PC_{18}H_{15})_4Cl_4](Na)^{2+}$
**3b**	2410.4472	2410.2191	94.639	$[Au_{13}(C_{31}H_{28}N_4)_3(PC_{18}H_{15})_3Cl_3]^{2+}$
**1c**	4169.7432	4169.3474	94.921	$[Au_{11}(C_{23}H_{28}N_4)(PC_{18}H_{15})_4Cl_2]^+$
	4213.7001	4213.2979	95.451	$[Au_{11}(C_{23}H_{28}N_4)(PC_{18}H_{15})_4ClBr]^+$
	2067.3894	2067.1890	96.934	$[Au_{11}(C_{23}H_{28}N_4)(PC_{18}H_{15})_4Cl]^{2+}$

Table S1: List of peaks recorded in the Q-TOF HRMS analyses of NHC-PPh$_3$ AuNCs

1.3 Reaction monitoring by HRMS

1.3.1 Q-TOF HRMS analyses of experiment involving **a**

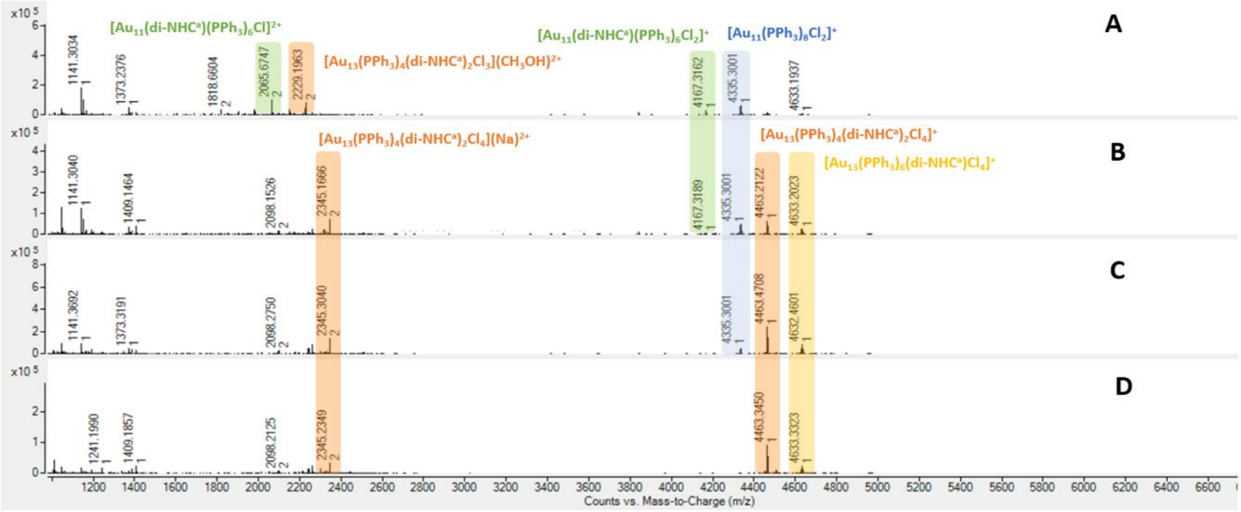

Figure S26: Q-TOF analyses of the reaction of $[Au_{11}(PPh_3)_8Cl_2]Cl$ with 2 equivalents of complex **a**. From above, after overnight warming (A), after 7 days (B), 14 days (C) and 21 days (D) at RT.

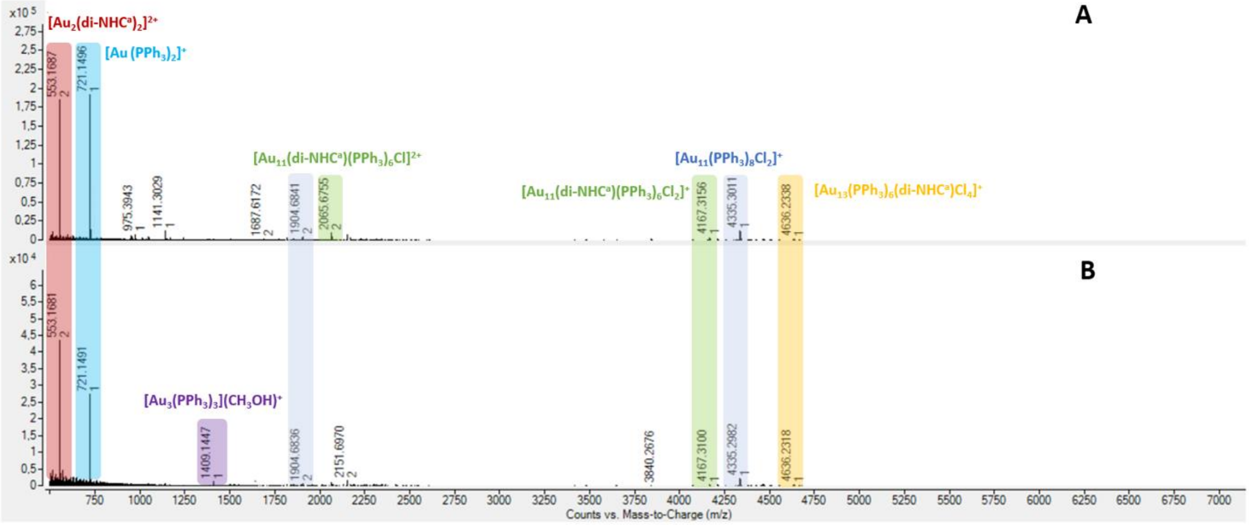

Figure S27: Q-TOF analyses of the reaction of $[Au_{11}(PPh_3)_8Cl_2]Cl$ with 3 equivalents of complex **a**. From above, after 21 h (A) and 48 h (B) under stirring at 40°C.

1.3.2 Q-TOF HRMS analyses of experiment involving **b**

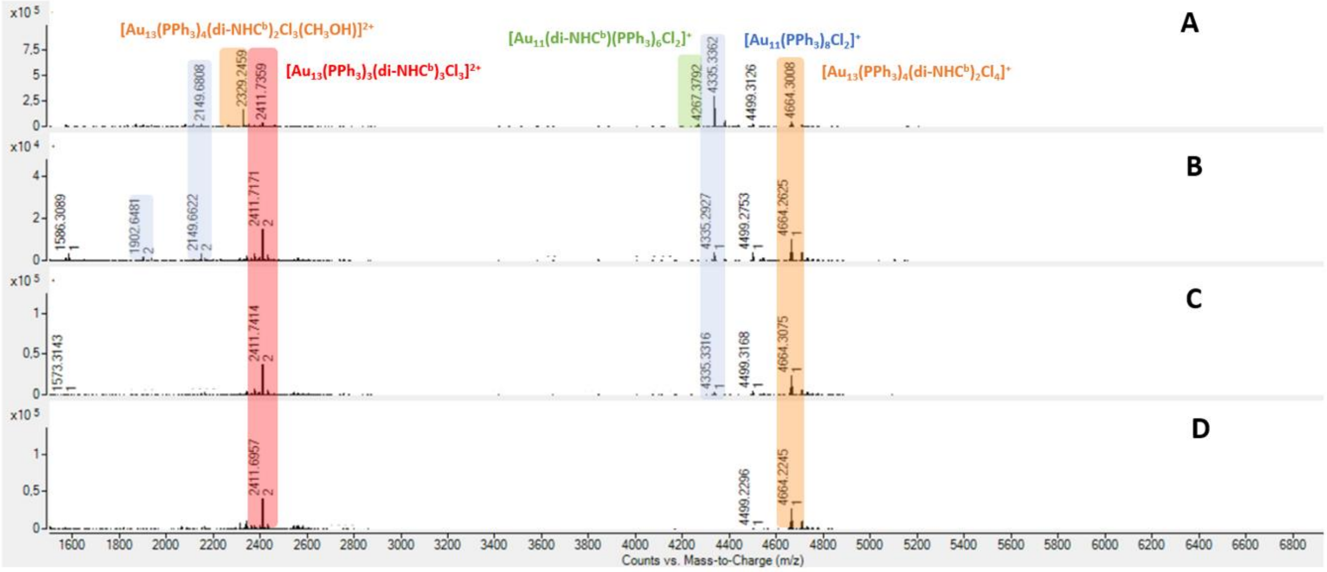

Figure S28: Q-TOF analyses of the reaction of $[Au_{11}(PPh_3)_8Cl_2]Cl$ with 2 equivalents of complex **b**. From above, after overnight warming (A), after 10 days (B), 15 days (C) and 17 days (D) at RT.

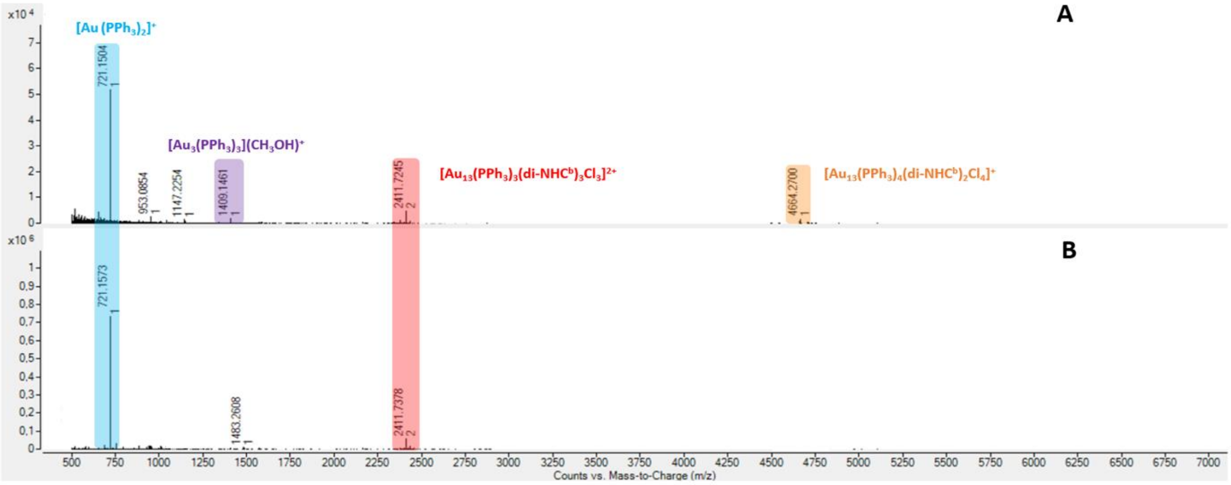

Figure S29: Q-TOF analyses of the reaction of $[Au_{11}(PPh_3)_8Cl_2]Cl$ with 3 equivalents of complex **b**. From above, after 3 days (A) and 5 days (B) under stirring at 40°C.

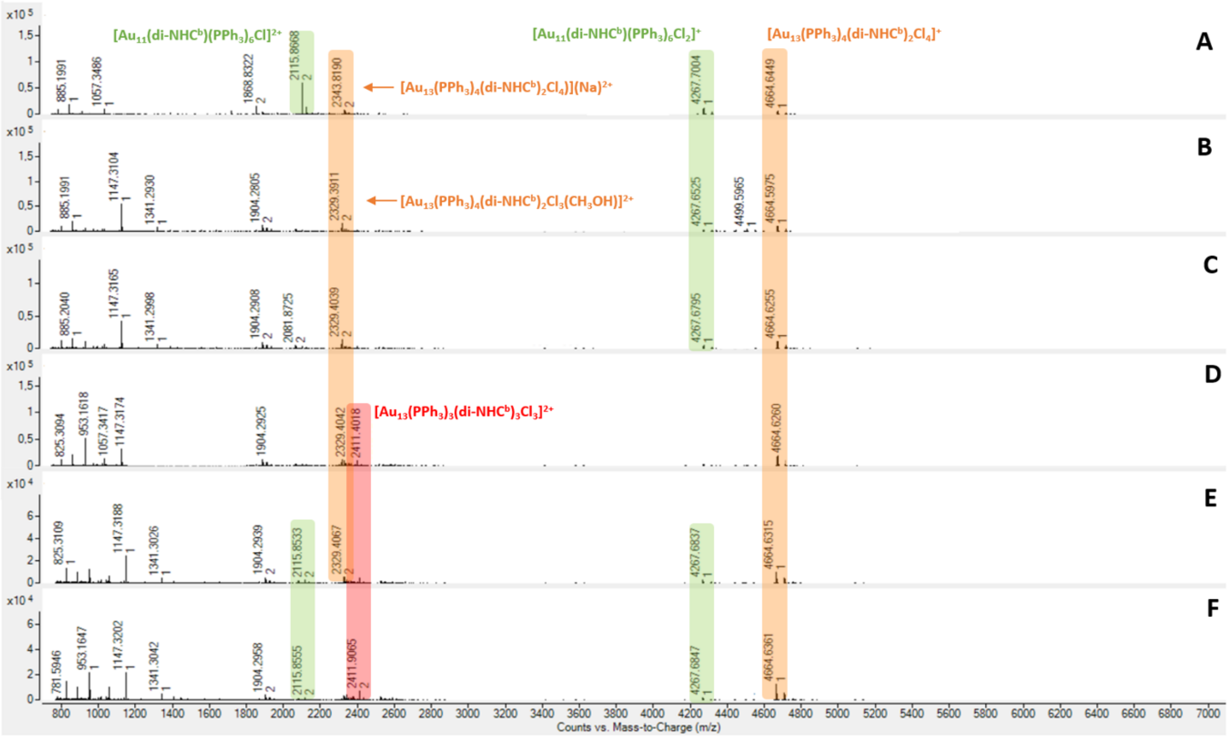

Figure S30: Q-TOF analyses of the reaction of **1b** with 1 equivalents of complex **b**, from above, after 0 h (A), 2 h (B), 17 h (C), 38 h (D), 50 h (E) and 72 h (F) under stirring at 40°C.

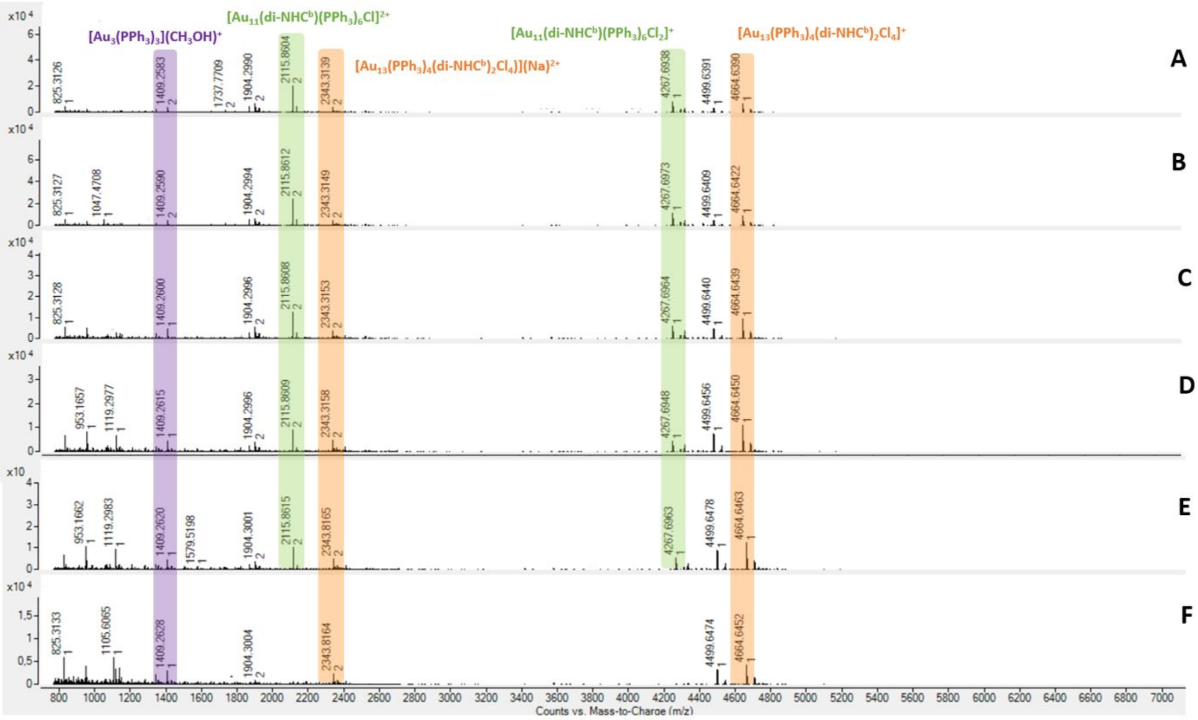

Figure S31: Q-TOF analyses of **1b** solution in DCM, from above, after 0 h (A), 2 h (B), 17 h (C), 38 h (D), 50 h (E) and 72 h (F) under stirring at 40°C.

1.3.3 Q-TOF HRMS analyses of experiment involving **c**

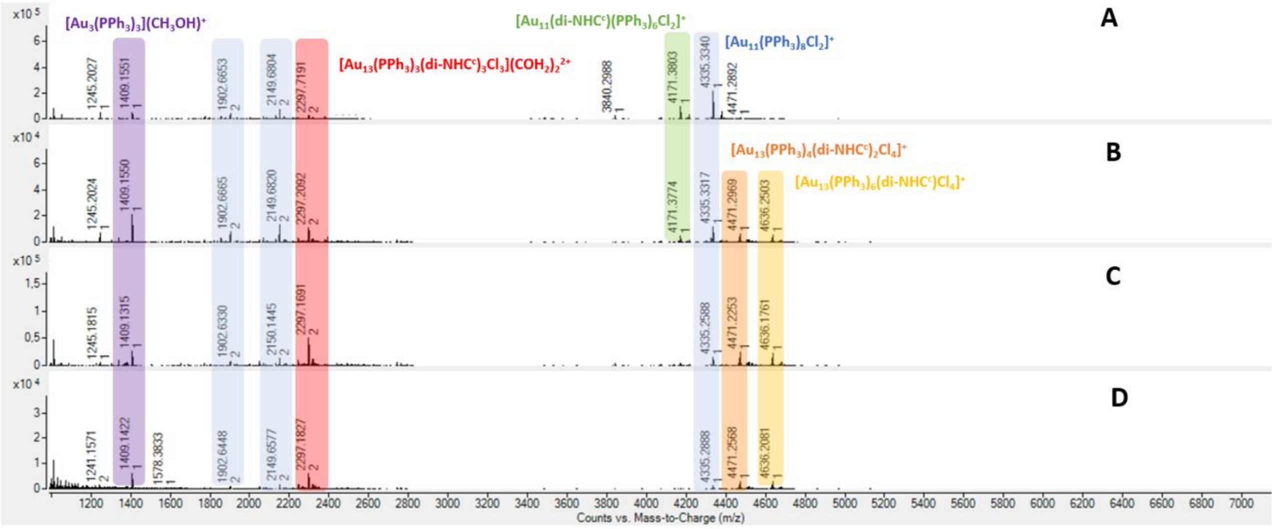

Figure S32: Q-TOF analyses of the reaction of $[Au_{11}(PPh_3)_8Cl_2]Cl$ with 2 equivalents of complex **c**. From above, after overnight warming (A), 15 days (B), 30 days (C) and 45 days (D) at RT.

1.4 UV-vis spectra of NHC-PPh_3 AuNCs

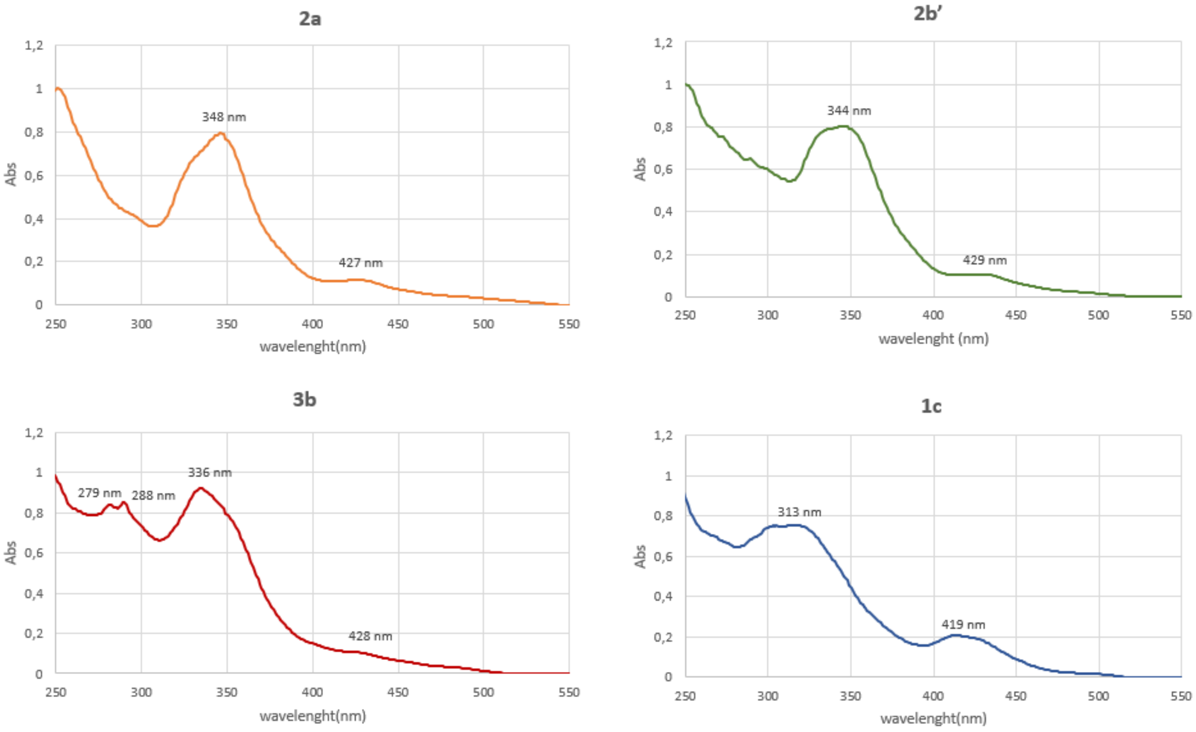

Figure S33: normalized UV-Vis spectra of **2a, 2b', 3b** and **1c** clusters in CH_2Cl_2. Maximum peaks are highlighted in the UV-vis spectra.

1.5 Emission spectra and QY value of NHC-PPh_3 AuNCs

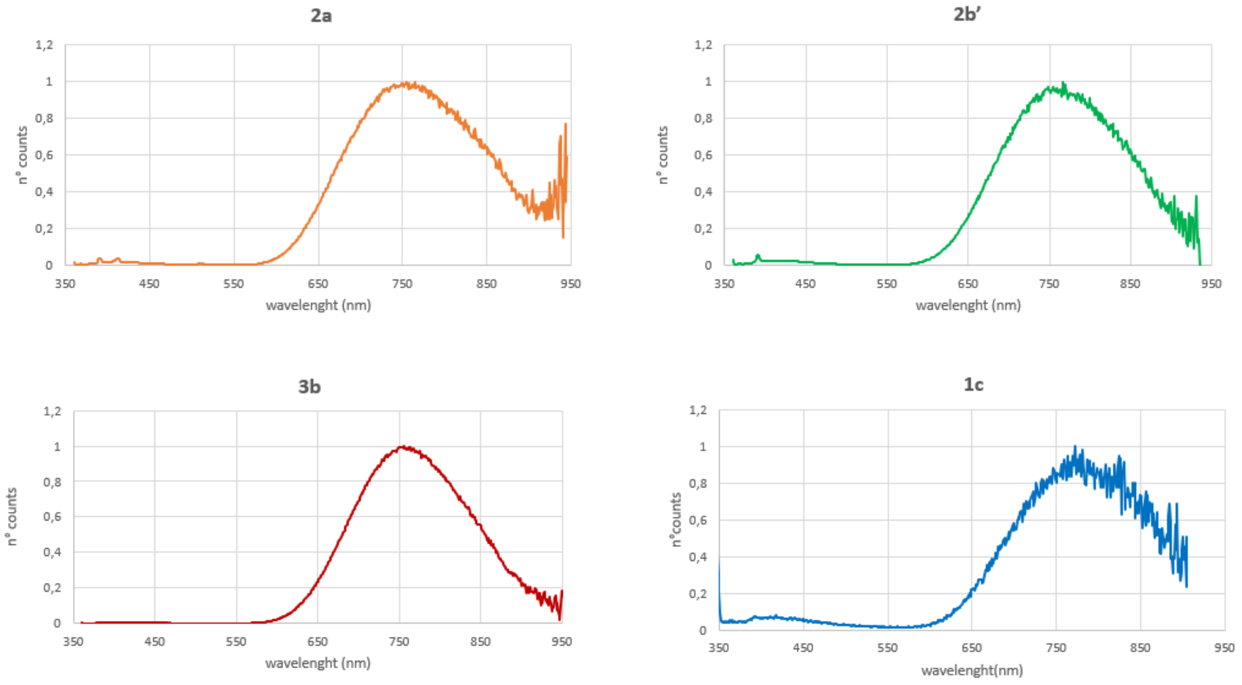

Figure S34: normalized emission spectra of **2a, 2b’, 3b** and **1c** clusters in CH_2Cl_2 (λ_{exc} : 350 nm; concentration: $2{\cdot}10^{-4}$ M, room temperature).

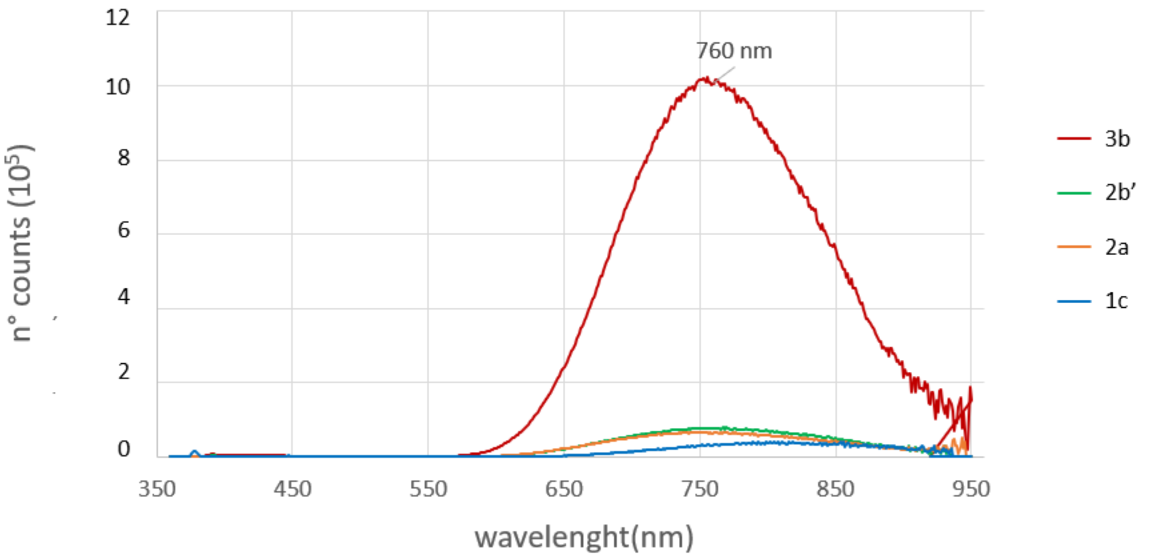

Figure S35: overlapped emission spectra of **2a, 2b’, 3b** and **1c** clusters in CH_2Cl_2 (λ_{exc} : 350 nm). All reported spectra are normalized upon absorbance value found at 350 nm in the UV-vis spectra.

Cluster	ε ($M^{-1}cm^{-1}$)	Quantum yield
**2a**	$4.0{\cdot}10^3$ (348 nm)	4.49 %
**2b'**	$2.3{\cdot}10^3$ (348 nm)	3.00 %
**3b**	$2.6{\cdot}10^3$ (336 nm)	44.46 %
**1c**	$4.0{\cdot}10^3$ (348 nm)	4.22 %

Table S2: Extinction coefficients and quantum yields of the studied AuNCs

1.6 X ray data collection for compound **3b**

The crystallographic data for compound 3b were obtained by mounting a well formed single crystal on a glass fiber and transferring it to a Bruker D8 Venture Photon II Bruker diffractometer. The APEX 3 program package[8] was used to obtain the unit-cell and the geometrical parameters and for the data collection. The raw frame data were processed using SAINT[1] and SADABS[2] to obtain the data file of the reflections. The structure was solved using SHELXT[3] (Intrinsic Phasing method in the APEX 3 program). The refinement of the structure (based on F2 by full-matrix least-squares techniques) was carried out using the SHELXTL-2014/7 program[4] in the WinGX suite v.2014.1. Detailed of the crystallographic data are reported in Table 2. Crystallographic data have been deposited with the Cambridge Crystallographic Data Centre as supplementary publication CCDC 2170527. Copies of the data can be obtained free of charge on application to the CCDC, 12 Union Road, Cambridge CB2 1EZ, U.K. (fax, (+44) 1223 336033; e-mail, deposit@ccdc.cam.ac.uk).

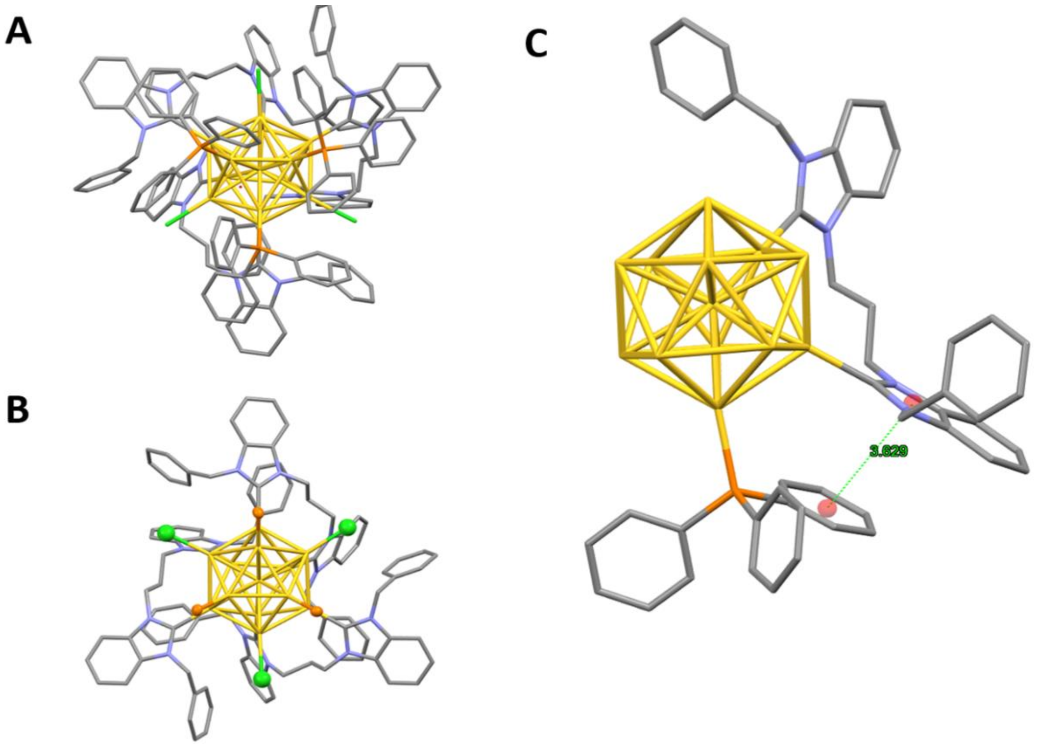

Figure S36: X-ray structure of **3b**. Full view of the cluster structure (A), full view after removal of the PPh_3 phenyl groups (B), π-π interaction among NHCs and PPh_3 ligands (C). Gold atoms are highlighted in yellow, chloride anions in green, phosphorous in orange, nitrogen in purple and carbon in grey. For clarity, hydrogens are omitted in all figures.

Formula	$[C_{147}H_{129}Au_{13}Cl_3N_{12}P_3]$ 7,5(CH_2Cl_2) Cl OH
Molecular weight	5512.84
Crystal system	Trigonal
Space group	R -3
a/Å	24.2867(3)
b/Å	24.2867(3)
c/Å	55.3721(10)
α/°	90.00
β/°	90.00
γ/°	120.00
Volume, $Å^3$	28285.1(9)
T (K)	200(2)
Z	3
D_{calc}/g cm^{-3}	1.942
F(000)	15354
⌠ (Mo-Kα)/mm^{-1}	10.41
Reflections collected	268625
Unique reflections	12884
Observed reflections [I>2⌠(*I*)]	11265 [R_{int} = 0.0653]
R [$I > 2\sigma(I)$]	R_1 = 0.0334<br>wR_2 = 0.1000
R [all data]	R_1 = 0.0404<br>wR_2 = 0.1083

$R_1 = \Sigma|Fo - Fc|/\Sigma|Fo|$; $wR_2 = [\Sigma[w(Fo^2 - Fc^2)^2]/\Sigma[w(Fo^2)^2]]^{1/2}$

Table S3: crystallographic data for **3b**

2.0 Bibliography

[1] Bruker. APEX3 and SAINT; Bruker AXS Inc.: Madison, WI, USA, **2015**.

[2] Krause, L.; Herbst-Irmer, R.; Sheldrick, G. M.; Stalke, D. Comparison of silver and molybdenum microfocus X-ray sources for single-crystal structure determination. *J. Appl. Crystallogr.* **2015**, *48*, 3–10.

[3] Sheldrick, G. M. Crystal structure refinement with SHELXL. *Acta Crystallogr., Sect. A: Found. Adv.* **2015**, *71*, 3–8.

[4] Farrugia, L. J. WinGX and ORTEP for Windows: an update. *J. Appl. Crystallogr.* **2012**, *45*, 849–854
